# Effects of probiotic administration on overweight or obese children: a meta-analysis and systematic review

**DOI:** 10.1186/s12967-023-04319-9

**Published:** 2023-08-04

**Authors:** Ya Li, Tonghua Liu, Lingling Qin, Lili Wu

**Affiliations:** Key Laboratory of Health Cultivation of Traditional Chinese Medicine, the Ministry of Education, Beijing University of Chinese Medicine, Beijing, 102488 China

**Keywords:** Probiotics, Overweight, Obesity, Children, Random control trials, Meta-analysis, Systematic review

## Abstract

**Background:**

This paper aimed to examine the effects of probiotics on eight factors in overweight or obese children by meta-analysis, namely, body mass index (BMI), total cholesterol (TC), triglyceride (TG), high-density lipoprotein cholesterol (HDL-C), low-density lipoprotein cholesterol (LDL-C), adiponectin, leptin and tumor necrosis factor-α (TNF-α) and summarize the mechanisms of action of probiotics based on the existing researches.

**Methods:**

Six databases (PubMed, Web of Science, Embase, Cochrane Library, SinoMed and CNKI) were searched until March 2023. Review Manager 5.4 was used for meta-analysis. The data were analysed using weighted mean differences (WMDs) or standardized mean differences (SMDs) under a fixed effect model or random effect model to observe the effects of probiotic administration on the included indicators.

**Results:**

Four publications with a total of 206 overweight or obesity children were included. According to the meta-analysis, probiotics were able to significantly decrease the levels of HDL-C (MD, 0.06; 95% CI 0.03, 0.09; P = 0.0001), LDL-C (MD, − 0.06; 95% CI − 0.12, − 0.00; P = 0.04), adiponectin (MD, 1.39; 95% CI 1.19, 1.59; P < 0.00001), leptin (MD, − 2.72; 95% CI − 2.9, − 2.54; P < 0.00001) and TNF-α (MD, − 4.91; 95% CI − 7.15, − 2.67; P < 0.0001) compared to those in the placebo group. Still, for BMI, the palcebo group seemed to be better than the probiotic group (MD, 0.85; 95% CI 0.04, 1.66; P = 0.04). TC (MD, − 0.05; 95% CI − 0.12, 0.02; P = 0.14) and TG (MD, − 0.16; 95% CI − 0.36, 0.05; P = 0.14) were not different between two groups.

**Conclusions:**

This review drew that probiotics might act as a role in regulating HDL-C, LDL-C, adiponectin, leptin and TNF-α in overweight or obesity children. Additionally, our systematic review yielded that probiotics might regulate lipid metabolism and improve obese associated symptoms by some paths. This meta-analysis has been registered at PROSPERO with ID: CRD42023408359.

**Supplementary Information:**

The online version contains supplementary material available at 10.1186/s12967-023-04319-9.

## Background

The prevalence of overweight or obesity in children is increasing due to changes in dietary structure and exercise habits, as determined by the body mass index (BMI) calculated from height and weight, with a BMI between the 85th and 94th percentile being classified as overweight and greater than the 94th percentile being classified as obese, of which 95% are simple obesity [1]. Since 1980, the global prevalence of childhood obesity has increased by 47.1% and has surpassed the growth rate of adult obesity. The rate of overweight and obesity among children aged 7 years and older in China is expected to reach 28% by 2030 [2]. Childhood obesity can cause some clinical complications such as hypertension, nonalcoholic fatty liver disease (NAFLD), and cardiovascular disease [3, 4]. In addition, it can also give rise to psychological problems such as low self-esteem [5, 6] depression, and even reduce the quality of life and shorten life expectancy [7].

The main causes of overweight or obesity in children are poor dietary habits and lack of exercise, which result in greater energy intake than consumption and lead to the accumulation of body fat [8]. Poor dietary behaviors include high consumption of sugary drinks and foods that are low in nutrients and high in saturated fat. The World Health Organization (WHO) recommends that children should have at least 60 min of moderate to vigorous physical activity each day, yet many children are not up to the standard amount of exercise [9, 10]. In addition, some unhealthy behaviors such as lack of sleep, excessive viewing of electronics and exposure to junk food advertising are also closely correlated with childhood obesity [11, 12]. The complex multifactorial etiology of the disease poses many challenges for researchers and clinicians in prevention and management.

The current treatment of overweight or obesity in children is mainly through the following three ways: First, to establish a healthy lifestyle [13]. Eat three regular meals and reduce eating out. Specifically, consume more fruits, vegetables, whole grains, protein and low-fat dairy products, while limiting the consumption of sugary drinks, sodium, solid fats and added sugars [14]. Ensure adequate physical activity and sleeping time, and also limit the time spent on electronic screens [15]. The second is pharmacological intervention. Orlistat, the only Food and Drug Administration-approved drug for the treatment of obesity in children 12 years and older [16, 17]. Third, bariatric surgery. Patients with BMI ≥ 35 kg/m^2^ with type 2 diabetes, moderate to severe sleep apnoea or severe NAFLD may consider bariatric surgery, which can reduce weight and alleviate associated complications [18].

However, all of the above approaches have their own drawbacks. For example, lifestyle changes lack personal self-control and external supervision, and drug therapy has limited usage due to significant gastrointestinal side impacts and not yet fully established effects. At the same time, surgical treatment requires multidisciplinary cooperation and delicate postoperative care. Therefore, there is still a need to find a safe and effective way to treat overweight or obesity in children.

Significant differences in gut microbiota between fat and lean children, which may be due to a high-fat and high-sugar diet that reduces intestinal flora diversity, particularly the relative abundance of butyric acid-producing bacteria and other beneficial flora [19]. In contrast, a rising body of studies have shown that gut flora can regulate energy metabolism, inflammation and immune regulation in the body [20, 21]. Furthermore, the gut microbiota is more vulnerable in childhood than in adulthood. Therefore, early intervention for the gut flora of overweight or obese children is essential to improve childhood health and lifetime gut microbiota homeostasis [22]. As defined by the WHO, probiotics are live microorganisms that regulate intestinal microecology and when given in adequate doses exert health benefits on the human body. The more commonly used are *Lactobacillus spp.* and *Bifidobacterium spp.*[23]. Some studies have also shown that probiotics can attenuate overweight or obesity in children [24]. However, other studies presented that probiotics are ineffective and even *Lactobacillus* species could increase weight [25, 26]. Given this, this study was conducted to explore whether probiotics could reduce overweight or obesity in children by a meta-analysis and further generalized its specific action mechanisms in a systematic review.

This paper adopted the PICO principle, including participants, intervention, comparison, and outcome. The specific factors are as follows: P—overweight or obese children; I—probiotics supplementation and unlimited types and forms; C—equal doses of placebo; and O—primary indicators of BMI, TC and TG, and secondary indicators of HDL-C, LDL-C, leptin, adiponectin, and TNF-α.

## Materials and methods

### Search strategy

This meta-analysis and systematic review were performed according to the Preferred Reporting Items for Systematic Reviews and Meta-Analyses (PRISMA) statement [27].

Six databases (PubMed, Embase, Cochrane Library, Web of Science, SinoMed, and CNKI [China National Knowledge Infrastructure]) were searched from inception to March 2023. The search terms were as follows: [(obese adolescents) OR (Pediatric Obesity) OR (Obesity, Pediatric) OR (Obesity in Childhood) OR (Childhood Onset Obesity) OR (Obesity, Childhood Onset) OR (Child Obesity) OR (Obesity, Child) OR (Childhood Obesity) OR (Obesity, Childhood) OR (Adolescent Overweight) OR (Overweight, Adolescent) OR (Infant Overweight) OR (Overweight, Infant) OR (Adolescent Obesity) OR (Obesity, Adolescent) OR (Obesity in Adolescence) OR (Childhood Overweight) OR (Overweight, Childhood) OR (Infantile Obesity) OR (Obesity, Infantile) OR (Infant Obesity) OR (Obesity, Infant)] AND [(probiotic agent) OR (gastrointestinal microbiota) OR (gut dysbiosis) OR (gut microbiota) OR (gut microbiome) OR (Probiotics)] AND [(randomized controlled trial OR randomized OR placebo)]. And then a total of 424 articles were searched, corresponding to n = 20, 25, 211, 159, 5, and 4 for the above databases (All articles searched from each database are shown in an Additional file 1: Table S1).

### Study selection

**Fig. 1:**
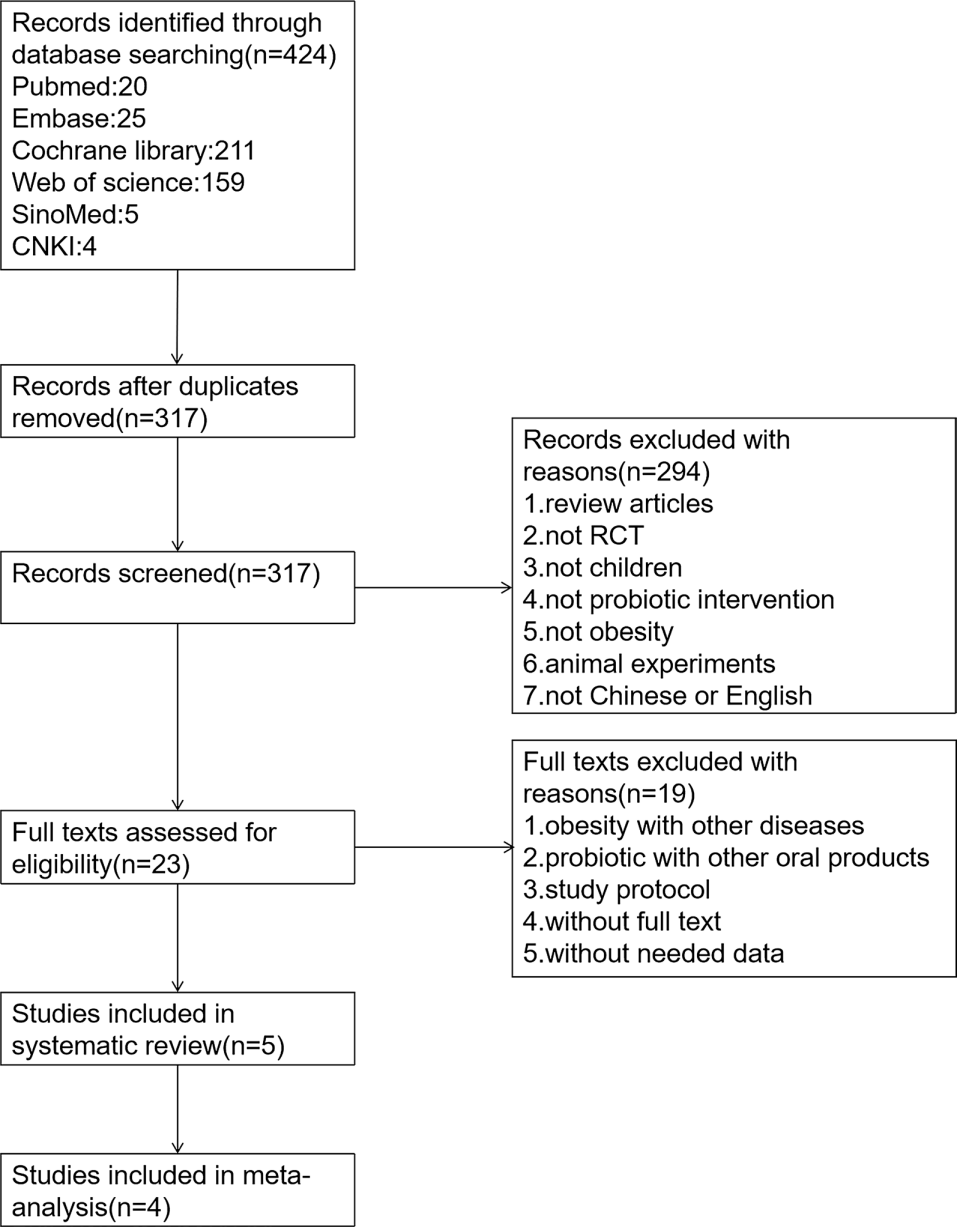
Study screening flowchart

Only randomized controlled trials of probiotics for overweight or obesity children were selected. Among them, the age range for children was 6–18 years old, and overweight or obesity children were free of anyother diseases. Nevertheless, articles of the following types were excluded: study protocols, full text unavailable, and no required data. The study screening process is shown in Fig. 1 (Specific review annotations for remaining 317 articles after removing duplicates are shown in an Additional file 1: Table S2).

### Data extraction

For the meta-analysis, the following information was summarized: (1) first author's name and publication year; (2) probiotics or placebo group, number of people in each group, and age range; and (3) different intervention methods of probiotic and placebo, intervention duration, and (4) outcomes observed.

For the systematic review, the following related information on the included studies was summarized: (1) first author’s name and year of publication; (2) strain types, oral forms and doses in probiotic group; (3) observation indicators; (4) efficacy factors and action mechanisms involved.

### Study quality assessment

**Fig. 2:**
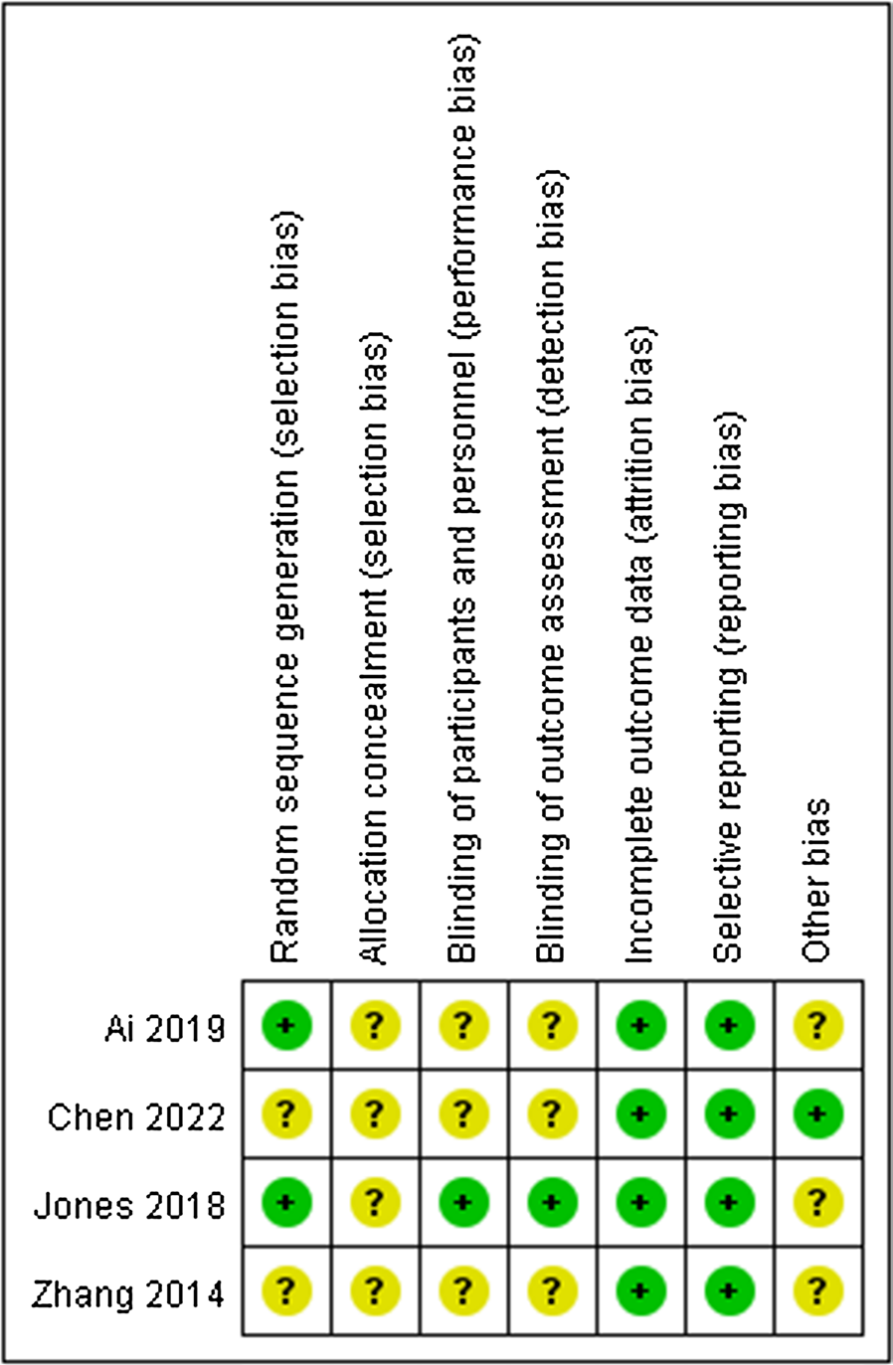
Risk of bias summary

The assessment of risk bias for the included studies was based on the evaluation criteria of the Cochrane Handbook [28]. This work was completed by two independent reviewers, and a third was responsible for resolving controversial issues. The risk bias assessment of the included studies was presented in Fig. 2.

### Observation outcomes

The primary outcomes in this study were BMI, TG, and TC, and the secondary outcomes included HDL-C, LDL-C, adiponectin, leptin and TNF-α.

### Data synthesis and analysis

A tool from the National Center for Biotechnology Information was used to complete the extraction of the graphical data for the included studies, and then Review Manager 5.4, a software for meta-analysis from the Cochrane Collaborative Network [29], was used for data synthesis and analysis. SMDs were selected if the units and the measurement methods of the observed indicators differed between studies; vice versa, WMDs were used [30]. 95% confidence intervals were applied to continuous variables. *P* value < 0.05 presented a statistical difference. Heterogeneity of articles was assessed based on I^2^ statistics, which is a percentage to quantify the magnitude of effect size variation in the study, and I^2^ of 0–25%, 25–50%, and 50–75% corresponding respectively to mild, moderate, and severe heterogeneity [31]. A fixed-effects model was adopted for data pooling when the I^2^ statistic was lower than 50%, representing an acceptable heterogeneity among the included studies. Conversely, a random-effects model was used [32].

## Results

### Study description in the meta-analysis

This meta-analysis included four studies with a total of 206 overweight or obesity childhood. Among them, 105 were in the probiotic group, and 101 were in the placebo group. The probiotic groups in these studies all used probiotic mixtures as interventions. Besides, only one study had an observation period of 15 days, whereas the others were longer than 2 months. Details are presented in Table 1.

**Table 1:** Specific characteristics of four articles included in the meta-analysis

Study	Groups (probiotics/placebo)	Age (years)	Interventions		Duration	Outcomes
			Probiotics	Placebo		
Ai [33]	54 (30/24)	6–14	Probiotic blends	Exercise and diet guidance	3 months	(1)(2)(8)
Chen et al. [34]	53 (27/26)	6–18	Functional ingredients and probiotics blends	Functional ingredients without probiotics blends	3 months	(1)(3)(4)(5)(6)(7)(8)
Jones et al. [25]	19 (8/11)	12–18	VSL#3®(probiotic blends)	Inactive product	16 weeks	(1)
Zhang et al. [35]	80 (40/40)	–	Probiotic blends	Regular diet	15 days	(1)(2)(3)(4)(5)(6)(7)

(1) = BMI; (2) = TG; (3) = TC; (4) = HDL; (5) = LDL; (6) = Leptin; (7) = Adiponectin; (8) = TNF-α

### Effects of probiotics on primary outcomes

**Fig. 3:**
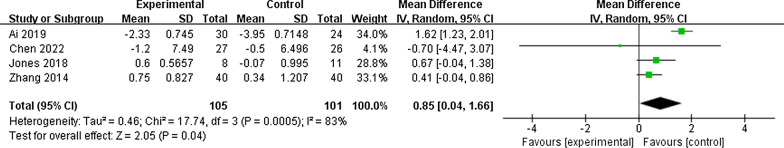
Forest plot of the effect of probiotics on BMI

**Fig. 4:**
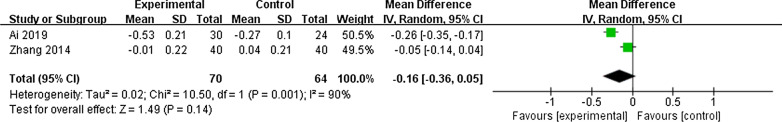
Forest plot of the effect of probiotics on TG

**Fig. 5:**
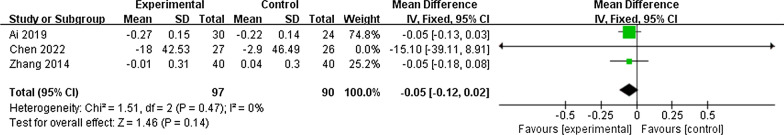
Forest plot of the effect of probiotics on TC

Four studies reported BMI (Fig. 3). The probiotic group was not as effective as the placebo group (WMD, 0.85; 95% CI 0.04, 1.66; P = 0.04). Significant heterogeneity was found (I^2^ = 83%, P = 0.0005). Two studies mentioned TG (Fig. 4). No statistically significant difference was observed between the two groups (WMD, − 0.16; 95% CI − 0.36, 0.05; P = 0.14). Remarkable heterogeneity was found (I^2^ = 90%, P = 0.001). Three studies covered TC (Fig. 5). No statistical differences were found between the two groups (WMD, − 0.05; 95% CI − 0.12, 0.02; P = 0.14). No heterogeneity was detected between the two groups (I^2^ = 0%, P = 0.47).

### Effect of probiotics on secondary outcomes

**Fig. 6:**
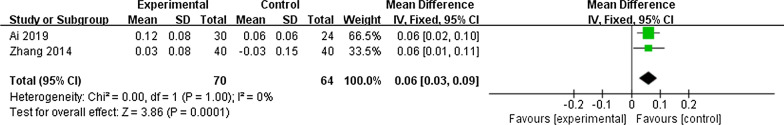
Forest plot of the effect of probiotics on HDL-C

**Fig. 7:**
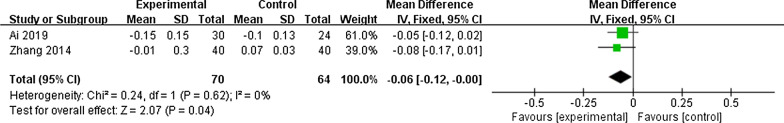
Forest plot of the effect of probiotics on LDL-C

**Fig. 8:**
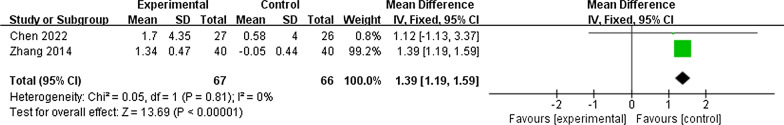
Forest plot of the effect of probiotics on adiponectin

**Fig. 9:**
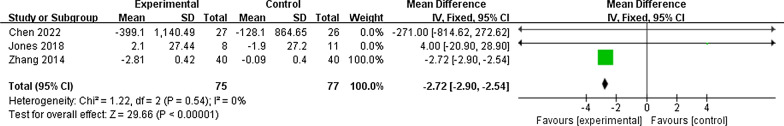
Forest plot of the effect of probiotics on leptin

**Fig. 10:**
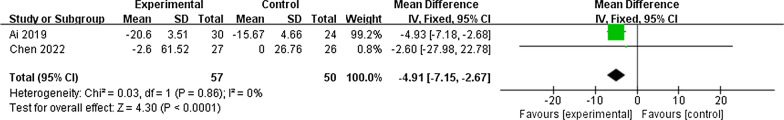
Forest plot of the effect of probiotics on TNF-α

Two studies contained HDL-C (Fig. 6). Significant difference was found between the two groups, and there was no heterogeneity (MD, 0.06; 95% CI 0.03, 0.09; P = 0.0001; I^2^ = 0%, P = 1.00 for heterogeneity). Two studies used the LDL-C (Fig. 7). The probiotic group was more efficacious than the placebo group (MD, − 0.06; 95% CI − 0.12, − 0.00; P = 0.04). No heterogeneity was observed. Two articles examined adiponectin (Fig. 8). The probiotic group was noticeably more efficient than the placebo group (MD, 1.39; 95% CI 1.19, 1.59; P < 0.00001). No heterogeneity was observed. Three articles addressed leptin (Fig. 9). The probiotic group was more efficient than the placebo group and no heterogeneity (MD, − 2.72; 95% CI − 2.9, − 2.54; P < 0.00001; I^2^ = 0%, P = 0.54). Two studies involved TNF-α (Fig. 10). A better result was in the probiotic group than the placebo group and no heterogeneity (MD, − 4.91; 95% CI − 7.15, − 2.67; P < 0.0001; I^2^ = 0%, P = 0.86 for heterogeneity).

### Probiotic mechanisms of action involved in included study

From this systematic review, we concluded that probiotics could improve overweight or obesity and related problems in children in some aspects. Ai et al. [33] found that probiotics could improve glycolipid metabolism and chronic inflammation related to obesity. Specifically, first, probiotics can lower cholesterol and improve blood lipid levels. Secondly, probiotics are able to enhance insulin sensitivity and improve blood glucose metabolism by protecting pancreatic beta cells, reducing endoplasmic reticulum stress and inhibiting macrophage activation. Finally, probiotics are also engaged in regulating immunity and metabolism processes to improve obesity related inflammation. Chen et al. [34] found that probiotics and functional ingredients might act synergistically to regulate the disordered intestinal flora, increase the abundance of *Lactobacillus spp.* and inhibit ether lipid metabolism. Also, it also reduced leptin and TNF-α levels and increased the secretion of adiponectin. Zhang et al. [35] discovered that probiotics could reduce chronic inflammation in the intestinal tract by regulating intestinal immune function, thereby affecting leptin and adiponectin levels, which in turn promotes body metabolism for weight loss. Larsen et al. [36] administered *Lactobacillus salivarius Ls-33* to obese adolescents and showed changes in the ratio of intestinal strains, inferring that *Ls-33* might act a regulatory activity on the intestinal microbiota. More details is in Table 2.

**Table 2:** Specific characteristics of five articles included in the systematic review

Study	Probiotics		Doses	Observation Indicators	Efficacy Factors	Action Mechanisms
	Types	Forms				
Ai [33]	*Bifidobacterium longum*; *Lactobacillus bulgaricus*; *Streptococcus thermophilus*	Tablets	6 g/d	BMI↓; TG↓; FBG↓; INS↓; HOMA-IR↓; IL-6↓ TNF-α↓; LBP↓	TLRs, NF-κB	Improving glycolipid metabolism and chronic inflammation
Chen et al. [34]	*Lactobacillus salivarius AP-32*; *Lactobacillus rhamnosus bv-77*; *Bififidobacterium animalis CP-9*	Packages	Thrss packages daily	HDL↑; adiponectin↑;BMI↓;TC↓; LDL↓; leptin↓; TNF-α↓; *Lactobacillus spp.*↑; *B. animalis*↑	Adipokines, NF-κB	Synergy between probiotics; Ether lipid metabolism; Regulation of the intestinal microbiota
Jones et al. [25]	–	Noncaloric, sugar-free flavoured drinks (vitaminwater zero)	Three times daily	BMI↑; total adiposity ↑; trunk adiposity↑	–	Altering functional potential of the gut microbiota
Zhang et al. [35]	*Bifidobacterium*; *Lactobacillus*; *Streptococcus thermophilus*	–	–	adiponectin↑; leptin↓;	Treg lymphocytes, Adipo R1, Adipo R2	Regulating intestinal immune function; Reducing chronic inflammation of the intestinal tract; Promoting energy metabolism
Larsen et al. [36]	*L.salivarius Ls-33 ATCC SD5208*	Capsule	One capsule daily	Ratios of *Bacteroidese*-*Prevotellae*-*Porphyromonas* group to *Firmicutes* belonging bacteria↑	–	Changing intestinal microbiota

*FBG* fasting blood glucose, *INS* insulin, *HOMA-IR* homeostatic model assessment of insulin resistance, *IL-6* interleukin-6, *LBP* lipopolysaccharide binding protein, *TLRs* toll-like receptors, *AdipoR* adiponectin receptor

## Discussion

From this meta-analysis, we found that probiotics could substantially improve HDL-C, LDL-C, adiponectin, leptin, TNF-α in overweight or obese children. Yet, there was no significant effect on BMI, TC and TG. The above results demonstrated that probiotics could regulate lipid metabolism and immune response to some degree in overweight or obese children. Based on this point, we continued to systematically review the mechanisms of action of probiotics on overweight or obese children.

However, probiotics mechanisms of action in regulating obesity is not fully understood and might be relevant to regulating intestinal flora homeostasis, improving energy metabolism and alleviating inflammation and immune response, etc.

1. Probiotics could reshape disturbed intestinal microbiota

Overweight or obesity is associated with a disturbed gut microbiota, with increased abundance of Bacteroides fragilis (B. fragilis), Escherichia coli (E. coli), Firmicutes and Staphylococci, and decreased abundance of Bifidobacteria, Desulfovibrio and Lactobacillus [34, 37, 38]. Gut flora in overweight or obese individuals could consume more food energy to gain weight, and might also negatively affect intestinal hormones [39, 40]. Even certain important intestinal strains, such as Thetaiotamicron bacteria and Methanobrevibacter smithii, could even promote fat accumulation [41]. However, Larsen et al. found an increase of the ratios of Bacteroides-Prevotella-Porphyromonas group to the Firmicutes belonging bacterial groups after children took Lactobacillus salivarius Ls-33 for 12 weeks, indicating that Ls-33 has regulatory activity on the intestinal microbiota [36]. And, in other studies it was also observed that probiotic supplements are able to increase the number of Bifidobacteria and short chain fatty acid (SCFA) producing Lactobacilli in the intestine. Conjugated linoleic acid produced by Bifidobacterium spp. and Lactobacillus spp. can promote lipolysis [42]. In addition, probiotics increased the abundance of Akkermansia muciniphila, which promotes the secretion of intestinal epithelial mucus, strengthens the intestinal barrier, reduces serum lipopolysaccharide (LPS) levels, TC, TG and LDL-C levels, and increases HDL-C [43]. Chen et al. [34] found that oral administration of probiotics increased the intestinal levels of Lactobacillus spp. and B. animalis in obese children, of which Lactobacillus spp. negatively associated with TC, LDL-C and etheric lipid metabolism but positively associated with HDL-C, and B. animalis positively associated with HDL and serum adiponectin levels and negatively associated with inflammation levels. Furthermore, low levels of B. fragilis and E. coli were observed in the probiotic group, and their abundance was positively correlated with obesity.

2. Probiotics regulate lipid metabolism

Overweight or obesity is the result of excessive lipid accumulation, where a high fat and high calorie diet causes lipid production to exceed lipolysis [44]. Yet, probiotics could inhibit lipid accumulation by improving lipid metabolism. Specifically, first, improving serum lipid profile. For example, a probiotic mixture containing *Lactobacillus salivarius AP-32, Lactobacillus rhamnosus bv-77* and *Bififidobacterium animalis CP-9* was able to reduce serum TC and LDL-C levels, markedly raise HDL-C level [34]. Second, regulating the expression of lipid related molecules. Such as *L. plantarum HAC01* was able to downregulate the expression of adipogenesis related genes, i.e. fatty acid synthase and stearoyl-CoA desaturase [45]. *B. breve B-3* increased expression of angiopoietin-like protein 4, which can inhibit lipoprotein lipase to reduce fat accumulation [46]. *L. kefiri* could increase the expression of peroxisome proliferator-activated receptor-α (PPAR-α), followed by upregulation of the expression of fatty acidbinding protein 4 (FABP4) and carnitine palmitoyltransferase-1 (CPT-1). Of which, FABP4 is an extracellular protein that inhibits lipogenesis and promotes lipolysis, and CPT-1 is a rate-limiting enzyme for fatty acid oxidation, catalyzing β-oxidation of fatty acids to the mitochondrial matrix. *L. sakei OK67* enhances the expression of adenosine monophosphate kinase (AMPK), which activates CPT-1 to promote mitochondrial fatty acid β-oxidation, and downregulates acetyl-CoA carboxylase and sterol regulatory element-binding protein-1c (SREBP-1c) to inhibit fatty acid synthesis [47–51]. Third, probiotics enzymatically dissolve bile acids. *Lactobacilli* and *Bifidobacteria* strains can produce bile salt hydrolase, which can hydrolyze bound bile acids into free bile acids, uncoupled bile acids are difficult to be reabsorbed in the intestine and mostly excreted with feces. This promotes cholesterol catabolism to synthesize more bile acids and thus lowering cholesterol level [52].

In addition, bile acids are able to regulate lipid levels by farnesoid X receptor (FXR) and secondary bile acids. On the one hand, the binding of bile acids to FXR inhibits the expression of SREBP-1c, subsequently prevents the synthesis of TG by fatty acid synthase, and also reduces very low density lipoprotein levels. Also, FXR increases fatty acid oxidation by raising the expression of PPAR-α activated receptors [53, 54]. On the other hand, secondary bile acids produced by the interaction of primary bile acids and intestinal flora are ligands for the G 5 protein-coupled receptor, reducing TG levels in the liver [55].

3. Probiotics could reduce inflammation and immune response

The types of cells in adipose tissue are different in fat and lean subjects. In lean subjects, adipose tissue mainly contains Treg lymphocytes, Th2 lymphocytes, eosinophils, and M2 macrophages while in obese individuals, it mainly includes CD8 + Tc, CD4 + Th1 and M1 macrophages. Proinflammatory macrophage infiltration in adipose tissue increases in higher levels of obesity, and adipocytes secrete more proinflammatory factors such as leptin, visfactin, resistin, which activate nuclear factor kappa-B (NF-κB) and activator protein 1, thus producing more inflammatory factors such as TNF-α and interleukin-6, triggering obesity-related inflammation [56–58]. Probiotics could decrease the production of pro-inflammatory factors and increase anti-inflammatory factors secreted by adipocytes. For example, both studies observd a significant decrease in leptin or TNF-α level and an increase in adiponectin levels after children were administered a probiotic mixture [34, 35]. Adiponection, an inflammation suppressor, can inhibit toll-like receptor 4-induced NF-κB activation and differentiate macrophages toward the anti-inflammatory M2 phenotype [59]. Apart from this, in some animal experiments, it was found that probiotics could regulate the immune response and relieve inflammation level. *B. animalis subsp. lactis Bi1*, *B. breve Bbr 8* and *B. breve BL10* could reduce the number of CD4 + T cells, total macrophages and activated M1 macrophage and lower levels of TNF-α and interferon-γ by suppressing NF-κB activation, but increase the production of interleukin-10 and transforming growth factor-β [60]. *L. rhamnosus LMG S-28148* and *B. animalis subsp. lactis LMG P-28149* downregulated the expressions of macrophage-specific genes CD68 and F4/80 and increased the level of regulatory T cell marker FOXP3 in adipose tissue, demonstrating that probiotics could promote the infiltration of Treg cells into adipose tissue and indirectly inhibit metabolic inflammation [61].

4. Probiotics could reduce inflammation and immune response

SCFAs are metabolites produced by probiotics fermenting and breaking down starch, indigestible polysaccharides, cellulose, non-fibrous polysaccharides, and mucin in the intestine [62, 63]. First of all, SCFAs are able to lower the ph value of intestinal microecology and inhibit the growth of pathogenic bacteria that are suitable for a weak alkaline environment. In addition, SCFAs play an important role in regulating the energy metabolism and immune response of the body after entering the systemic circulation as a molecular signal. For one, SCFAs can interact with G protein-coupled receptor (GPR) 41 and GPR43 to increase the expression of polypeptide YY and glucagon-like peptide 1 in the intestine, which stimulates changes in cortical neuronal excitability, enhances satiety and reduces fat accumulation [64]. Second, SCFAs activate AMPK in the liver and muscle, increasing fatty acid oxidation [65]. Acetic acid could also increase the expression of mitochondrial lipid oxidation genes [66]. Third, acetic acid, propionic acid and butyric acid, which account for more than 95% of the SCFA, are able to alleviate the low-grade inflammation associated with metabolic diseases by inhibiting NF-κB activity and reducing local macrophage infiltration to decrease the production of pro-inflammatory cytokines and chemokines [67]. Fourth, SCFAs could induce the secretion of tight junction proteins and mucins in the intestinal mucosa to enhance the integrity of the intestinal epithelial barrier, thus reducing the outward infiltration of LPS in the intestinal lumen, and preventing the occurrence of the inflammatory cascade [68].

5. Probiotics alleviate oxidative stress and endoplasmic reticulum stress

Chronic high sugar and high fat diets produce excess reactive oxygen species (ROS), leading to oxidative stress, which is characterized by greater oxidative capacity than antioxidant effects. Severe oxidative stress is an important factor in the pathogenesis of obesity. In detail, excess ROS could cause lipid peroxidation that decreases mitochondrial membrane electrical potential and causes mitochondrial dysfunction, which in turn the reduction of fatty acid β-oxidation, increase in TG and impaired normal lipid metabolism [69]. It was found that *LAB* has the ability to scavenge free radicals and inhibit lipid peroxidation [70]. *L. coryniformis subsp. torquens T3* and *L. paracasei subsp. paracasei M5* can increase the level of glutathione peroxidase, catalase and total superoxide dismutase and reduce the content of lipid peroxidation product malondialdehyde in obese persons [71]. Endoplasmic reticulum stress, namely, agglutination of unfolded proteins, is also a factor in the pathogenesis of obesity. *L. plantarum MTCC5690* and *L. fermentum MTCC5689* alleviated the high fat diet induced increase in endoplasmic reticulum stress markers such as GRP78, PERK, and IRE1α, etc. in skeletal muscle [72]. Oxidative stress and endoplasmic reticulum stress could also result to inflammatory responses through activation of TNF-α, NF-κB and JNK signaling pathways. *L. plantarum MTCC5690*, *L. fermentum MTCC5689* and *L. rhamnosus GG* could alleviate inflammation level caused by endoplasmic reticulum stress [73].

6. Probiotic other pathways of action

In addition, probiotics could also inhibit the growth and proliferation of pathogens in the intestine by competitive exclusion and colonization resistance of metabolites, etc. On the one hand, probiotics could inhibit pathogens growth by competing for receptor sites and nutrients in the intestine. For example, *Escherichia coli Nissle 1917* and *E. coli HS* supressed the growth of pathogenic *E. coli* by competing for the same carbohydrates [74]. On the other hand, probiotics can also produce bacteriocins, an antimicrobial peptide consisting of 30–60 amino acids, which prevent the proliferation of some pathogens. For instance, *L. plantarum* and *L. acidophilus* produce antimicrobial peptides that inhibit the growth and proliferation of *Helicobacter pylori*, *C. diffcile*, rotaviruses, multidrug-resistant *Shigella spp.* and *E. coli* [75]. Reuterin produced by *L. reuteri* could inhibit the reproduction of *Helicobacter pylori* and *C. difficile* so that protects the gastrointestinal tract [76]. With the development of sequencing technology, more and more beneficial bacteria in the intestinal microecology are being identified, such as *B. fragilis* with the ability to inhibit pathogenic bacterial infection [77].

In this study, we found that probiotics could reduce overweight or obesity and related health problems in children somewhat by a variety of approaches. This study might serve as a more reliable reference for subsequent research works and feasible future clinical applications. Nevertheless, our study still has some limitations. Firstly, the number of randomized controlled trials examining the effect of probiotics on overweight or obese children is small and the sample size of the included study is limited. Secondly, most probiotic administrations are in the form of mixtures, whereas the effect of different probiotic strains on overweight or obesity might be different, there is a lack of research on the effect of single probiotic strain. Thirdly, the short observation period of one of the included articles, combined with the poor compliance of children during the clinical study, might have caused some bias to the results.

Further more clinical studies are still needed to conduct. In the first place, to determine factors such as more beneficial probiotic strains, optimal intake forms and appropriate doses in order to understand the exact relationship between probiotics and overweight or obese children. In addition, some basic experiments are need to explore the specific mechanisms of action of probiotics in improving overweight or obesity related health problems, enabling us to gain a deeper insight into the role of probiotics.

## Conclusions

In this meta-analysis, we found that probiotics can improve HDL-C, LDL-C, adiponectin, leptin, and TNF-α in overweight or obese children, therefore we examined the mechanisms of action of probiotics through a systematic review, and found that probiotics work mainly by reshaping disturbed intestinal microbiota, regulating lipid metabolism, reducing inflammation and immune response, playing a positive effect of SCFAs produced, alleviating oxidative stress and endoplasmic reticulum stress, and inhibiting the growth and reproduction of pathogens in the gut.

### Supplementary Information

**Additional file 1: Table S1.** All articles searched from each database. **Table S2.** Specific review annotations for remaining 317 articles after removing duplicates.

## Data Availability

The original data involved in the manuscript can be obtained from references.

## Abbreviations

BMI: Body mass index
TC: Total cholesterol
TG: Triglyceride
HDL-C: High-density lipoprotein cholesterol
LDL-C: Low-density lipoprotein cholesterol
TNF-α: Tumor necrosis factor-α
WMD: Weighted mean difference
SMD: Standardized mean difference
NAFLD: Nonalcoholic fatty liver disease
WHO: World Health Organization
SCFA: Short chain fatty acid
LPS: Lipopolysaccharide
FABP4: Fatty acidbinding protein 4
CPT-1: Carnitine palmitoyltransferase-1
AMPK: Adenosine monophosphate kinase
SREBP-1c: Sterol regulatory element-binding protein-1c
FXR: Farnesoid X receptor
NF-κB: Activate nuclear factor kappa-B
IL-6: Interleukin-6
GPR: G protein-coupled receptor
ROS: Reactive oxygen species
