# Supplementary material for: Effects of probiotic administration on overweight or obese children: a meta-analysis and systematic review
Source: J Transl Med. 2023 Aug 4;21:525. doi: 10.1186/s12967-023-04319-9 (PMC10401801; doi:10.1186/s12967-023-04319-9)
Supplement: Supplementary file 1 — Additional file 1: Table S1. All articles searched from each database. Table S2. Specific review annotations for remaining 317 articles after removing duplicates. [file 12967_2023_4319_MOESM1_ESM.docx]

**Table S1.** All articles searched from each database.

| Databases | Articles Searched |
| --- | --- |
| PubMed （20） | 1. [Effects of Fecal Microbiome Transfer in Adolescents With Obesity: The Gut Bugs Randomized Controlled Trial.](https://pubmed.ncbi.nlm.nih.gov/33346848/) |
|  | 1. Therapeutic Effects of Butyrate on Pediatric Obesity: A Randomized Clinical Trial. |
|  | 1. [Maternal bacteria to correct abnormal gut microbiota in babies born by C-section.](https://pubmed.ncbi.nlm.nih.gov/32791721/) |
|  | 1. [Exercise training modulates the gut microbiota profile and impairs inflammatory signaling pathways in obese children.](https://pubmed.ncbi.nlm.nih.gov/32624568/) |
|  | 1. [Prebiotics Reduce Body Fat and Alter Intestinal Microbiota in Children Who Are Overweight or With Obesity.](https://pubmed.ncbi.nlm.nih.gov/28596023/) |
|  | 1. [Effects of inulin supplementation on body composition and metabolic outcomes in children with obesity.](https://pubmed.ncbi.nlm.nih.gov/35906473/) |
|  | 1. [Supplementation with Bifidobacterium breve BR03 and B632 strains improved insulin sensitivity in children and adolescents with obesity in a cross-over, randomized double-blind placebo-controlled trial.](https://pubmed.ncbi.nlm.nih.gov/34229263/) |
|  | 1. Gut Microbiota and Obesity in Adults and Children: The State of the Art. |
|  | 1. [A time-restricted feeding intervention in children and adolescents with obesity: The TRansForm study protocol.](https://pubmed.ncbi.nlm.nih.gov/36386926/) |
|  | 1. A multi-strain probiotic blend reshaped obesity-related gut dysbiosis and improved lipid metabolism in obese children. |
|  | 1. [Characterization of the Stool Microbiome in Hispanic Preschool Children by Weight Status and Time.](https://pubmed.ncbi.nlm.nih.gov/29028448/) |
|  | 1. The Macronutrient Composition of Infant Formula Produces Differences in Gut Microbiota Maturation That Associate with Weight Gain Velocity and Weight Status. |
|  | 1. [Effects of probiotics combined with dietary and lifestyle modification on clinical, biochemical, and radiological parameters in obese children with nonalcoholic fatty liver disease/nonalcoholic steatohepatitis: a randomized clinical trial.](https://pubmed.ncbi.nlm.nih.gov/34773939/) |
|  | 1. [Delivery mode-associated gut microbiota in the first 3 months of life in a country with high obesity rates: A descriptive study.](https://pubmed.ncbi.nlm.nih.gov/33019428/) |
|  | 1. [Evaluation of the gut microbiota after metformin intervention in children with obesity: A metagenomic study of a randomized controlled trial.](https://pubmed.ncbi.nlm.nih.gov/33360047/) |
|  | 1. [Protocol for the Gut Bugs Trial: a randomised double-blind placebo-controlled trial of gut microbiome transfer for the treatment of obesity in adolescents.](https://pubmed.ncbi.nlm.nih.gov/31005929/) |
|  | 1. Probiotic supplementation increases obesity with no detectable effects on liver fat or gut microbiota in obese Hispanic adolescents: a 16-week, randomized, placebo-controlled trial. |
|  | 1. [Study Protocol of a Multicenter Randomized Controlled Trial to Tackle Obesity through a Mediterranean Diet vs. a Traditional Low-Fat Diet in Adolescents: The MED4Youth Study.](https://pubmed.ncbi.nlm.nih.gov/34062731/) |
|  | 1. Effects of synbiotic supplementation on anthropometric indices and body composition in overweight or obese children and adolescents: a randomized, double-blind, placebo-controlled clinical trial. |
|  | 1. [Evaluation of differential effects of metformin treatment in obese children according to pubertal stage and genetic variations: study protocol for a randomized controlled trial.](https://pubmed.ncbi.nlm.nih.gov/27432166/) |
| Embase（25） | 1. Maternal bacteria to correct abnormal gut microbiota in babies born by C-section |
|  | 1. Effect of probiotics intake on obese children |
|  | 1. Effects of Butyrate Against Pediatric Obesity: The BAPO randomized-controlled trial |
|  | 1. Efficacy of metformin and fermentable fiber combination therapy in adolescents with severe obesity and insulin resistance: study protocol for a double-blind randomized controlled trial |
|  | 1. Effects of probiotics on nonalcoholic fatty liver disease in obese children and adolescents |
|  | 1. Gut microbiome biomarkers in adolescent obesity: A regional study |
|  | 1. Effect of probiotics and dietary changes on adiposity in children |
|  | 1. Gut microbiota and BMI throughout childhood: the role of firmicutes, bacteroidetes, and short-chain fatty acid producers |
|  | 1. A randomized triple-masked controlled trial on the effects of synbiotics on inflammation markers in overweight children |
|  | 1. A randomized triple-masked controlled trial on the effects of synbiotics on inflammation markers in overweight children |
|  | 1. Effects of synbiotic supplementation on anthropometric measurements, glucose metabolism, lipid parameters in children with exogenous obesity: A randomized, double blind, placebocontrolled clinical trial (probesity-2 trial) |
|  | 1. Effects of Fecal Microbiome Transfer in Adolescents with Obesity: The Gut Bugs Randomized Controlled Trial |
|  | 1. Probiotic therapy outcomes in body composition of children and adolescent with obesity, a nonrandomized controlled trial |
|  | 1. Effect of prebiotic fiber-induced changes in gut microbiota on adiposity in obese and overweight children |
|  | 1. Prebiotics Reduce Body Fat and Alter Intestinal Microbiota in Children Who Are Overweight or With Obesity |
|  | 1. Effect of prebiotic fiber intake on adiposity and inflammation in overweight and obese children: Assessing the role of the gut microbiota |
|  | 1. Evaluation of differential effects of metformin treatment in obese children according to pubertal stage and genetic variations: Study protocol for a randomized controlled trial |
|  | 1. Evaluation of the gut microbiota after metformin intervention in children with obesity: A metagenomic study of a randomized controlled trial |
|  | 1. Effects of probiotics combined with dietary and lifestyle modification on clinical, biochemical, and radiological parameters in obese children with nonalcoholic fatty liver disease/nonalcoholic steatohepatitis: a randomized clinical trial |
|  | 1. Supplementation with Bifidobacterium breve BR03 and B632 strains improved insulin sensitivity in children and adolescents with obesity in a cross-over, randomized double-blind placebo-controlled trial |
|  | 1. Efficacy of the treatment with bifidobacterium breve b632 and bifidobacterium breve br03 on endocrine response to the oral glucose tolerance test in pediatric obesity: A cross-over double blind randomized controlled trial |
|  | 1. The effects of inulin supplementation on insulin resistance and branched-chain amino acids in paediatric obesity: A randomised, double-blinded, placebo-controlled trial |
|  | 1. Effects of inulin supplementation on body composition and metabolic outcomes in children with obesity |
|  | 1. Effects of inulin supplementation on gut microbiota in obese children: A randomised, doubleblinded placebo-controlled study |
|  | 1. Greater alteration of gut microbiota occurs in childhood obesity than adulthood obesity |
| Cochrane library  （211） | 1. Gut Bugs Trial - Gut microbiome transfer for the treatment of adolescent obesity |
|  | 1. Vegetables as first foods for babies study |
|  | 1. Hydrolyzed Formula With Reduced Protein Content Supports Adequate Growth: a Randomized Controlled Noninferiority Trial |
|  | 1. Randomised clinical trial: the beneficial effects of VSL#3 in obese children with non-alcoholic steatohepatitis |
|  | 1. Effects of Bifidobacterium animalis Subsp. lactis (BPL1) Supplementation in Children and Adolescents with Prader-Willi Syndrome: a Randomized Crossover Trial |
|  | 1. Nutrition Society of New Zealand Annual Conference |
|  | 1. Effect of probiotic yoghurt on plasma glucose in overweight and obese pregnant women: a randomized controlled clinical trial |
|  | 1. A Vegetarian Diet Is a Major Determinant of Gut Microbiota Composition in Early Pregnancy |
|  | 1. Effects of underfeeding and oral vancomycin on gut microbiome and nutrient absorption in humans |
|  | 1. The usefulness of probiotic administration in obese children with nonalcoholic fatty liver disease |
|  | 1. Beneficial effects of Bifidobacterium lactis on lipid profile and cytokines in patients with metabolic syndrome: a randomized trial. Effects of probiotics on metabolic syndrome |
|  | 1. Exploring the Impact of Child-Centered Play Therapy on Academic Achievement of At-Risk Kindergarten Students |
|  | 1. Study protocol of a multicenter randomized controlled trial to tackle obesity through a mediterranean diet vs. A traditional low-fat diet in adolescents: the med4youth study |
|  | 1. Probiotic supplementation and trimethylamine-N-oxide production following a high-fat diet |
|  | 1. Therapeutic microbiology: the role of bifidobacterium breve as food supplement for the prevention/treatment of paediatric diseases |
|  | 1. Maternal bacteria to correct abnormal gut microbiota in babies born by C-section |
|  | 1. Probiotics for the Prevention of Gestational Diabetes Mellitus in Overweight and Obese Women: findings From the SPRING Double-Blind Randomized Controlled Trial |
|  | 1. Effect of probiotics on postoperative quality of gastric bypass surgeries: a prospective randomized trial |
|  | 1. A synbiotic mixture of scGOS/lcFOS and Bifidobacterium breve M-16V is able to restore the delayed colonization of bifidobacterium observed in C-section Delivered Infants |
|  | 1. Effect of pre/probiotic mixture in children with nonalcoholic fatty liver disease: a randomized controlled trial |
|  | 1. Impact of probiotic feeding during weaning on the serum lipid profile and plasma metabolome in infants |
|  | 1. Microbial enterotypes beyond genus level: bacteroides species as a predictive biomarker for weight change upon controlled intervention with arabinoxylan oligosaccharides in overweight subjects |
|  | 1. Intermittent fasting in experimental autoimmune encephalomyelitis and multiple sclerosis |
|  | 1. Prebiotics in infant nutrition |
|  | 1. Therapeutic Effects of Butyrate on Pediatric Obesity: a Randomized Clinical Trial |
|  | 1. Effects of Butyrate Against Pediatric Obesity: the BAPO randomized-controlled trial |
|  | 1. Genetic Polymorphisms, Mediterranean Diet and Microbiota-Associated Urolithin Metabotypes can Predict Obesity in Childhood-Adolescence |
|  | 1. Probiotics and Maternal Mental Health: a Randomised Controlled Trial among Pregnant Women with Obesity |
|  | 1. A new possible therapeutic strategy in obesityrelated liver disease: preliminary data with lactobacillus rhamnosus strain GG |
|  | 1. Efficacy of metformin and fermentable fiber combination therapy in adolescents with severe obesity and insulin resistance: study protocol for a double-blind randomized controlled trial |
|  | 1. Do athletes' responses to coach autonomy support and control depend on the situation and athletes' personal motivation? |
|  | 1. Serum metabolite profiling yields insights into health promoting effect of A. muciniphila in human volunteers with a metabolic syndrome |
|  | 1. Wheat bran with reduced particle size increases serum SCFAs in obese subjects without improving health parameters compared with a maltodextrin placebo |
|  | 1. Insight into the prebiotic concept: lessons from an exploratory, double blind intervention study with inulin-type fructans in obese women |
|  | 1. Regular exercise attenuates inflammatory signaling pathways and modulates metabolomic profile in gut microbiota from obese paediatric patients |
|  | 1. Beneficial effects of aerobic and resistance combined training on inflammatory signaling pathways and gut microbiota in obese pediatric patients |
|  | 1. Comparison of Ursodeoxycholic Acid to Metformin to treat women with Gestational Diabetes Mellitus |
|  | 1. Effects of Probiotics on Nonalcoholic Fatty Liver Disease in Obese Children and Adolescents |
|  | 1. Impact of wheat aleurone on biomarkers of cardiovascular disease, gut microbiota and metabolites in adults with high body mass index: a double-blind, placebo-controlled, randomized clinical trial |
|  | 1. PHAGE Study: effects of Supplemental Bacteriophage Intake on Inflammation and Gut Microbiota in Healthy Adults |
|  | 1. Human Milk Oligosaccharides Modulate Fecal Microbiota and Are Safe for Use in Children With Overweight: a Randomized Controlled Trial |
|  | 1. Body Mass Index and Gut Microbiome: a Cluster-Randomized, Controlled, Pilot Feasibility Study |
|  | 1. Gut microbiota in adolescent girls with polycystic ovary syndrome: effects of randomized treatments |
|  | 1. A randomised controlled trial of a duodenal-jejunal bypass sleeve device (EndoBarrier) compared with standard medical therapy for the management of obese subjects with type 2 diabetes mellitus |
|  | 1. Probiotics to adolescents with obesity: effects on inflammation and metabolic syndrome |
|  | 1. Nutritional Intervention Preconception and During Pregnancy to Maintain Healthy Glucose Metabolism and Offspring Health ("NiPPeR"): study protocol for a randomised controlled trial |
|  | 1. Improvement of chronic inflammation in obese children consecutive to probiotic intake |
|  | 1. Multistrain probiotic increases the gut microbiota diversity in obese pregnant women: results from a randomized, double-blind placebo-controlled study |
|  | 1. Effects of probiotics (Vivomixx®) in obese pregnant women and their newborn: study protocol for a randomized controlled trial |
|  | 1. Multi-strain probiotic increases the gut microbiot a diversity in obese pregnant women: results from a randomized, double-blind placebo-controlled study |
|  | 1. Effect of probiotics and dietary changes on adiposity in children |
|  | 1. Effect of probiotics and dietary changes on inflammatory markers and adiposity in children |
|  | 1. Effect of case study versus video simulation on nursing students' satisfaction, self-confidence, and knowledge: a quasi-experimental study |
|  | 1. Link between gut microbiota and health outcomes in inulin -treated obese patients: lessons from the Food4Gut multicenter randomized placebo-controlled trial |
|  | 1. Facilitating a high-quality dietary pattern induces shared microbial responses linking diet quality, blood pressure, and microbial sterol metabolism in caregiver-child dyads |
|  | 1. Characterization of the Stool Microbiome in Hispanic Preschool Children by Weight Status and Time |
|  | 1. The Impacts of Fish Oil and/or Probiotic Intervention on Low-Grade Inflammation, IGFBP-1 and MMP-8 in Pregnancy: a Randomized, Placebo-Controlled, Double-Blind Clinical Trial |
|  | 1. The efficacy of probiotics and/or n-3 long-chain polyunsaturated fatty acids intervention on maternal prenatal and postnatal depressive and anxiety symptoms among overweight and obese women |
|  | 1. Low-protein formula slows weight gain in infants of overweight mothers |
|  | 1. Effects of synbiotic on anthropometry, lipid profile and oxidative stress in obese children |
|  | 1. The effect of low carbohydrate diet on food addiction and gut microflora |
|  | 1. Benefits of repeated individual dietary counselling in long-term weight control in women after delivery |
|  | 1. Effects of probiotic supplementation on gut microbiota and obesity outcomes in obese hispanic adolescents: a 16-week, randomized, placebo-controlled trial |
|  | 1. Probiotic supplementation increases obesity with no detectable effects on liver fat or gut microbiota in obese Hispanic adolescents: a 16-week, randomized, placebo-controlled trial |
|  | 1. Impact of probiotics during weaning on the metabolic and inflammatory profile: follow-up at school age |
|  | 1. Probiotics during weaning: a follow-up study on effects on body composition and metabolic markers at school age |
|  | 1. A randomized triple-masked controlled trial on the effects of synbiotics on inflammation markers in overweight children |
|  | 1. Arabinoxylan oligosaccharides and polyunsaturated fatty acid effects on gut microbiota and metabolic markers in overweight individuals with signs of metabolic syndrome: a randomized cross-over trial |
|  | 1. Protocol for a multicentre, parallel, randomised, controlled trial on the effect of sweeteners and sweetness enhancers on health, obesity and safety in overweight adults and children: the SWEET project |
|  | 1. Effect of alive probiotic on insulin resistance in type 2 diabetes patients: randomized clinical trial |
|  | 1. The Effects of Limosilactobacillus reuteri LR-99 Supplementation on Body Mass Index, Social Communication, Fine Motor Function, and Gut Microbiome Composition in Individuals with Prader-Willi Syndrome: a Randomized Double-Blinded Placebo-Controlled Trial |
|  | 1. Probiotic Bifidobacterium strains and galactooligosaccharides improve intestinal barrier function in obese adults but show no synergism when used together as synbiotics |
|  | 1. Evaluation of yellow pea fibre supplementation on weight loss and the gut microbiota: a randomized controlled trial |
|  | 1. Consuming yellow pea fiber reduces voluntary energy intake and body fat in overweight/obese adults in a 12-week randomized controlled trial |
|  | 1. Predictors of Session Attendance in Intimate Partner Violence Treatment for Trauma-Exposed Veterans |
|  | 1. Effect of Lactobacillus salivarius Ls-33 on fecal microbiota in obese adolescents |
|  | 1. A Synbiotic Mixture of Scgos/Lcfos and Bifidobacterium Breve M-16V Is Able to Restore the Delayed Colonization of Bifidobacterium Observed in C-Section Delivered Infants |
|  | 1. Effects of education and support groups organized by IBCLCs in early postpartum on breastfeeding |
|  | 1. Protocol for the Gut Bugs Trial: a randomised double-blind placebo-controlled trial of gut microbiome transfer for the treatment of obesity in adolescents |
|  | 1. Effects of Fecal Microbiome Transfer in Adolescents With Obesity: the Gut Bugs Randomized Controlled Trial |
|  | 1. Study protocol: optimized complementary feeding study (OTIS): a randomized controlled trial of the impact of a protein-reduced complementary diet based on Nordic foods |
|  | 1. Effects of vaginal seeding on gut microbiota, body mass index, and allergy risks in infants born through cesarean delivery: a randomized clinical trial |
|  | 1. Effects of Vaginal Seeding on Gut Microbiota, Body Mass Index, and Allergy Risks in Cesarean-Delivered Infants: a Randomized Clinical Trial |
|  | 1. Long-term safety and efficacy of perinatal probiotic intervention: evidence from a follow-up study of four randomized, double-blind, placebo-controlled trials |
|  | 1. Long-term safety and efficacy of perinatal probiotic intervention: evidence from a follow-up study of four randomized, double-blind, placebo-controlled trials |
|  | 1. The impact of perinatal probiotic intervention on the development of overweight and obesity: follow-up study from birth to 10 years |
|  | 1. Impact of maternal probiotic-supplemented dietary counselling on pregnancy outcome and prenatal and postnatal growth: a double-blind, placebo-controlled study |
|  | 1. A pilot study of synbiotic supplementation on breast milk mineral concentrations and growth of exclusively breast fed infants |
|  | 1. Six weeks of oral Echinacea purpurea supplementation does not enhance the production of serum erythropoietin or erythropoietic status in recreationally active males with above-average aerobic fitness |
|  | 1. An alcohol-free beer enriched with isomaltulose and a resistant dextrin modulates gut microbiome in subjects with type 2 diabetes mellitus and overweight or obesity: a pilot study |
|  | 1. Impact of dietary fiber supplementation on modulating microbiota-host-metabolic axes in obesity |
|  | 1. The Macronutrient Composition of Infant Formula Produces Differences in Gut Microbiota Maturation That Associate with Weight Gain Velocity and Weight Status |
|  | 1. The Macronutrient Composition of Infant Formula Produces Differences in Gut Microbiota Maturation That Associates with Weight Gain Velocity and Weight Status |
|  | 1. Primed to perform: comparing different pre-performance routine interventions to improve accuracy in closed, self-paced motor tasks |
|  | 1. Urinary (1)H-NMR-based metabolic profiling of children with NAFLD undergoing VSL#3 treatment |
|  | 1. The Effects of Probiotic Supplements on Blood Markers of Endotoxin and Lipid Peroxidation in Patients Undergoing Gastric Bypass Surgery; a Randomized, Double-Blind, Placebo-Controlled, Clinical Trial with 13 Months Follow-Up |
|  | 1. Metagenomics analysis of gut microbiota in response to diet intervention and gestational diabetes in overweight and obese women: a randomised, double-blind, placebo-controlled clinical trial |
|  | 1. Consumption of stilbenes and flavonoids is linked to reduced risk of obesity independently of fiber intake |
|  | 1. Probiotics reduce self-reported symptoms of upper respiratory tract infection in overweight and obese adults: should we be considering probiotics during viral pandemics? |
|  | 1. Daily probiotic use is associated with a reduced rate of upper respiratory tract symptoms in overweight and obese people |
|  | 1. The Effects of Synbiotic Supplementation on Antioxidant Capacity and Arm Volumes in Survivors of Breast Cancer-Related Lymphedema |
|  | 1. The Effect of Probiotics on Low-grade Inflammation, Microbiota and Risk Factors for Metabolic Syndrome in Obese Children |
|  | 1. Effects of Probiotics in Obese Children |
|  | 1. Effect of Prebiotic Intake on Adiposity, Satiety and Gut Microbiota in Overweight and Obese Children |
|  | 1. Potential Role of n-3 Fatty Acids in the Treatment of NAFLD in Pediatric Patients |
|  | 1. Potential Role of n-3 Fatty Acids in the Treatment of NAFLD in Pediatric Patients |
|  | 1. Growth and Metabolism in Infants Fed Protein-reduced, Alpha-lactalbumin Enriched Formula |
|  | 1. Effect of Probiotics (Vivomixx®) on Weight, Microbiota and Glucose Tolerance in Obese Pregnant Women and Their Newborn |
|  | 1. Transplantation of Microbes for Treatment of Metabolic Syndrome & NAFLD |
|  | 1. Preventing Atopic Dermatitis and ALLergies in Children |
|  | 1. The Effect of Lactobacillus Reuteri ATCC PTA 6475 on Volumetric Bone Mineral Density in Patients With Osteopenia |
|  | 1. Healthy Eating Through Reduction Of Excess Sugar |
|  | 1. Effects of HMOs on the Faecal Microbiota and on Host Metabolism in Obese Children |
|  | 1. The Effects of Butyrate on Children With Obesity |
|  | 1. Vaginal Microbiome Seeding and Health Outcomes in Cesarean-delivered Neonates |
|  | 1. BIFI-OBESE: clinical Trial in Paediatric Obesity |
|  | 1. Effects of Almond Consumption on Cardiovascular, Metabolomic, and Microbiome Profiles in Millennials |
|  | 1. Composition and Collection Feasibility of Gut Microbiota in Children With and Without Obesity |
|  | 1. Fit Child - Clinical Study on Weight Control |
|  | 1. Gut Microbiome, Adiposity, and Probiotics (GMAP) |
|  | 1. Dietary Management of Gestational Diabetes in Obese Pregnant Women |
|  | 1. Response of the Gut Microbiome and Circulating Metabolome to Diet in Children: ancillary Study to KIDFIT (NCT03405246) |
|  | 1. Dietary Management of Gestational Diabetes |
|  | 1. Targeting the Gut Microbiome for Prader-Willi Syndrome Treatment |
|  | 1. Motivational Intervention on the Gut Microbiota of Obese Children |
|  | 1. Strategies To OPpose Sugars With Non-nutritive Sweeteners Or Water (STOP Sugars NOW) Trial |
|  | 1. Impact of Dietary Fiber as Prebiotics on Intestinal Microbiota in Obese Thai Children |
|  | 1. Evaluating the Metabolic Effects of Polylactose: a Novel Prebiotic |
|  | 1. Impact of B. Bifidum 900791 Intake on Breast Milk Characteristics of Obese Mothers |
|  | 1. Effect of a Three Combined Probiotics Supplementation on Weight Loss in Obese/Overweight Children |
|  | 1. Effect of Liraglutide on Microbiome in Obesity |
|  | 1. Fiber Intervention on Gut Microbiota in Children With Prader-Willi Syndrome |
|  | 1. China Heart Diet for People With High Cardiovascular Risk in China (DECIDE-Diet) |
|  | 1. L. Reuteri DSM 17938 and L. Reuteri ATCC PTA 6475 in Moderate to Severe Irritable Bowel in Adults |
|  | 1. Assessment of the Impact of Type of Probiotic, Delivery Type and Feeding Type on Baby's Microbiota After Dysbiotic Delivery |
|  | 1. Fiber and Metformin Combination Therapy in Adolescents With Severe Obesity and Insulin Resistance |
|  | 1. Maternal Betaine Supplementation During Breastfeeding |
|  | 1. Effects of Butyrate Against Pediatric Obesity |
|  | 1. Probiotic Treatment for Prader-Willi Syndrome |
|  | 1. PROBESITY-2: synbiotics in Pediatric Obesity |
|  | 1. Childhood Obesity Microbiome Study |
|  | 1. The Influence of GNiib® in Obesity Management in Obese Young Individuals in Hong Kong |
|  | 1. Dietary Intervention With Probiotic Pasta and Evaluation of the Effects on Metabolic and Inflammatory Status |
|  | 1. Dietary Modulation of Gut Microbiota in Overweight/Obese Adolescents and COVID-19 Infection |
|  | 1. Time-Restricted Feeding in Children and Adolescents With Obesity |
|  | 1. Growth, Allergy and Neurodevelopment in Infants on Hydrolysed Formula |
|  | 1. Immune-supportive Diet and Gut Permeability in Allergic Children |
|  | 1. Comparison of Plant-based or Animal-based Protein on Anthropocentric and Metabolic Parameters in Obese Subjects |
|  | 1. Assessment of the Effects of Synbiotic on Gut Microbiota Composition in Scholars With Overweight |
|  | 1. Keiki (Pediatric) Produce Prescription (KPRx) Program Hawaii |
|  | 1. Full-fat Dairy Products, Body Weight Control and Metabolic Health |
|  | 1. Comparative Clinical Study Between Empagliflozin Versus Pioglitazone in Non-diabetic Patients With Non-alcoholic Steatohepatitis |
|  | 1. Prebiotics Reduce Body Fat and Alter Intestinal Microbiota in Children Who Are Overweight or With Obesity |
|  | 1. Prebiotics Reduce Body Fat and Alter Intestinal Microbiota in Children Who Are Overweight or With Obesity |
|  | 1. Effect of prebiotic fiber-induced changes in gut microbiota on adiposity in obese and overweight children |
|  | 1. Effect of prebiotic fiber intake on adiposity and inflammation in overweight and obese children: assessing the role of the gut microbiota |
|  | 1. SPRING: an RCT study of probiotics in the prevention of gestational diabetes mellitus in overweight and obese women |
|  | 1. Effect of antenatal dietary interventions in maternal obesity on pregnancy weight-gain and birthweight: healthy Mums and Babies (HUMBA) randomized trial |
|  | 1. A randomised controlled demonstration trial of multifaceted nutritional intervention and or probiotics: the healthy mums and babies (HUMBA) trial |
|  | 1. Correction: a randomised controlled demonstration trial of multifaceted nutritional intervention and or probiotics: the healthy mums and babies (HUMBA) trial [BMC Pregnancy Childbirth., 16, (2016) (373)] DOI: 10.1186/s12884-016-1149-8 |
|  | 1. Probiotic supplementation attenuates increases in body mass and fat mass during high-fat diet in healthy young adults |
|  | 1. Evaluation of differential effects of metformin treatment in obese children according to pubertal stage and genetic variations: study protocol for a randomized controlled trial |
|  | 1. Evaluation of the gut microbiota after metformin intervention in children with obesity: a metagenomic study of a randomized controlled trial |
|  | 1. Safety and tolerability of experimental hookworm infection in humans with metabolic disease: study protocol for a phase 1b randomised controlled clinical trial |
|  | 1. Gut microbiota (GM) and gut-liver axis (GLA) components in obesity related NAFLD: a pediatric pilot study |
|  | 1. Effects of School-Based Participation Program to Prevent Multiple Risk Behaviors in Thai Male Adolescents |
|  | 1. Antibiotic exposure in the first three years of life and weight gain during childhood |
|  | 1. Effect of Synbiotic Supplementation in Children with Nonalcoholic Fatty Liver Disease: a Randomized Controlled Trial |
|  | 1. Exercise training modulates the gut microbiota profile and impairs inflammatory signaling pathways in obese children |
|  | 1. Effect of probiotic Lactobacillus plantarum Dad-13 powder consumption on the gut microbiota and intestinal health of overweight adults |
|  | 1. Effects of bifidobacterium animalis subsp. lactis on children with Prader-Willi syndrome: a randomized, double-blind, placebo-controlled, crossover trial |
|  | 1. Improved Plasma Lipids, Anti-Inflammatory Activity, and Microbiome Shifts in Overweight Participants: two Clinical Studies on Oral Supplementation with Algal Sulfated Polysaccharide |
|  | 1. Effects of probiotics combined with dietary and lifestyle modification on clinical, biochemical, and radiological parameters in obese children with nonalcoholic fatty liver disease/nonalcoholic steatohepatitis: a randomized clinical trial |
|  | 1. The effects of synbiotic supplementation on some cardio-metabolic risk factors in overweight and obese children: a randomized triple-masked controlled trial |
|  | 1. Inulin-type fructans modulate intestinal Bifidobacterium species populations and decrease fecal short-chain fatty acids in obese women |
|  | 1. Reinforcement of intestinal epithelial barrier by arabinoxylans in overweight and obese subjects: a randomized controlled trial: arabinoxylans in gut barrier |
|  | 1. Effects of a Diet-Based Weight-Reducing Program with Probiotic Supplementation on Satiety Efficiency, Eating Behaviour Traits, and Psychosocial Behaviours in Obese Individuals |
|  | 1. Bifidobacterium pseudocatenulatum CECT 7765 supplementation improves inflammatory status in insulin-resistant obese children |
|  | 1. Bifidobacterium pseudocatenulatum CECT 7765 supplementation improves inflammatory status in insulin-resistant obese children |
|  | 1. Fish Oil And/Or Probiotics Intervention in Overweight/Obese Pregnant Women and Overweight Risk in 24-Month-Old Children |
|  | 1. Early Gut Fungal and Bacterial Microbiota and Childhood Growth |
|  | 1. Soy food intake associates with changes in the metabolome and reduced blood pressure in a gut microbiota dependent manner |
|  | 1. Prospective Longitudinal Trends in Body Composition and Clinical Outcomes 3 Years Following Sleeve Gastrectomy |
|  | 1. Multiomic Predictors of Short-Term Weight Loss and Clinical Outcomes During a Behavioral-Based Weight Loss Intervention |
|  | 1. Supplementation with Bifidobacterium breve BR03 and B632 strains improved insulin sensitivity in children and adolescents with obesity in a cross-over, randomized double-blind placebo-controlled trial |
|  | 1. Efficacy of the treatment with bifidobacterium breve b632 and bifidobacterium breve br03 on endocrine response to the oral glucose tolerance test in pediatric obesity: a cross-over double blind randomized controlled trial |
|  | 1. Efficacy of diode and CO2 lasers along with calcium and fluoride-containing compounds for the remineralization of primary teeth |
|  | 1. The impact of probiotics’ administration on glycemic control, body composition, gut microbiome, mitochondria, and other hormonal signals in adolescents with prediabetes – A randomized, controlled trial study protocol |
|  | 1. A maternal higher-complex carbohydrate diet increases bifidobacteria and alters early life acquisition of the infant microbiome in women with gestational diabetes mellitus |
|  | 1. A maternal higher-complex carbohydrate diet increases bifidobacteria and alters early life acquisition of the infant microbiome in women with gestational diabetes mellitus |
|  | 1. The Effectiveness of the Young–Old Link and Growth Intergenerational Program in Reducing Age Stereotypes |
|  | 1. The effect of synbiotic supplementation on anthropometric indices, appetite, and constipation in people with hypothyroidism: a randomized, double-blind, placebo-controlled trial |
|  | 1. Effects of Complementary Feeding With Different Protein-Rich Foods on Infant Growth and Gut Health: study Protocol |
|  | 1. Evaluation of the effect of Lactobacillus reuteri V3401 on biomarkers of inflammation, cardiovascular risk and liver steatosis in obese adults with metabolic syndrome: a randomized clinical trial (PROSIR) |
|  | 1. Structural Alteration of Gut Microbiota during the Amelioration of Human Type 2 Diabetes with Hyperlipidemia by Metformin and a Traditional Chinese Herbal Formula: a Multicenter, Randomized, Open Label Clinical Trial |
|  | 1. The effects of inulin supplementation on insulin resistance and branched-chain amino acids in paediatric obesity: a randomised, double-blinded, placebo-controlled trial |
|  | 1. Calorie restriction and synbiotics effect on quality of life and edema reduction in breast cancer-related lymphedema, a clinical trial |
|  | 1. The impact of probiotic supplementation during pregnancy on DNA methylation of obesity-related genes in mothers and their children |
|  | 1. Effects of Lactobacillus rhamnosus strain GG in pediatric obesity-related liver disease |
|  | 1. A randomized double-blind placebo controlled pilot study of probiotics in adolescents with severe obesity |
|  | 1. Effects of inulin supplementation on body composition and metabolic outcomes in children with obesity |
|  | 1. Effects of inulin supplementation on gut microbiota in obese children: a randomised, doubleblinded placebo-controlled study |
|  | 1. Metagenomic Insights into the Degradation of Resistant Starch by Human Gut Microbiota |
|  | 1. Role-play versus lecture methods in community health volunteers |
|  | 1. Strain engraftment competition and functional augmentation in a multi-donor fecal microbiota transplantation trial for obesity |
|  | 1. Genomic microdiversity of Bifidobacterium pseudocatenulatum underlying differential strain-level responses to dietary carbohydrate intervention |
|  | 1. Mendelian Randomization Analysis Reveals Causal Effects of the Human Gut Microbiota on Abdominal Obesity |
|  | 1. The mediating effect of coping styles and self‐efficacy between perceived stress and satisfaction with QOL in Chinese adolescents with type 1 diabetes |
|  | 1. A Quasi-Trial Investigation of an In-Service Training to Improve Social Workers' Professional Competence in China |
|  | 1. Metabolic phenotypes and the gut microbiota in response to dietary resistant starch type 2 in normal-weight subjects: a randomized crossover trial |
|  | 1. Evaluation of a WeChat‐based life review programme for cancer patients: a quasi‐experimental study |
| Web of Science  （159） | 1. Evaluation of the gut microbiota after metformin intervention in children with obesity: A metagenomic study of a randomized controlled trial |
|  | 1. [Maternal bacteria to correct abnormal gut microbiota in babies born by C-section](https://www.webofscience.com/wos/alldb/full-record/WOS:000558420900067) |
|  | 1. [The effects of synbiotic supplementation on some cardio-metabolic risk factors in overweight and obese children: a randomized triple-masked controlled trial](https://www.webofscience.com/wos/alldb/full-record/WOS:000322716400005) |
|  | 1. [Low-Protein Formula Slows Weight Gain in Infants of Overweight Mothers](https://www.webofscience.com/wos/alldb/full-record/WOS:000338125000026) |
|  | 1. [Supplementation with Bifidobacterium breve BR03 and B632 strains improved insulin sensitivity in children and adolescents with obesity in a cross-over, randomized double-blind placebo-controlled trial](https://www.webofscience.com/wos/alldb/full-record/WOS:000684395200010) |
|  | 1. [Protocol for the Gut Bugs Trial: a randomised double-blind placebo-controlled trial of gut microbiome transfer for the treatment of obesity in adolescents](https://www.webofscience.com/wos/alldb/full-record/WOS:000471157200169) |
|  | 1. [The impact of perinatal probiotic intervention on the development of overweight and obesity: follow-up study from birth to 10 years](https://www.webofscience.com/wos/alldb/full-record/WOS:000282727000012) |
|  | 1. [Three-year follow-up of a randomised controlled trial to reduce excessive weight gain in the first two years of life: protocol for the POI follow-up study](https://www.webofscience.com/wos/alldb/full-record/WOS:000381548200002) |
|  | 1. [Prebiotic supplementation improves appetite control in children with overweight and obesity: a randomized controlled trial](https://www.webofscience.com/wos/alldb/full-record/WOS:000398941700005) |
|  | 1. [The Macronutrient Composition of Infant Formula Produces Differences in Gut Microbiota Maturation That Associate with Weight Gain Velocity and Weight Status](https://www.webofscience.com/wos/alldb/full-record/WOS:000774274300001) |
|  | 1. [Probiotic supplementation increases obesity with no detectable effects on liver fat or gut microbiota in obese Hispanic adolescents: a 16-week, randomized, placebo-controlled trial](https://www.webofscience.com/wos/alldb/full-record/WOS:000449476600011) |
|  | 1. [Evaluation of differential effects of metformin treatment in obese children according to pubertal stage and genetic variations: study protocol for a randomized controlled trial](https://www.webofscience.com/wos/alldb/full-record/WOS:000379816600002) |
|  | 1. [Weight Gain and Obesity in Infants and Young Children Exposed to Prolonged Antibiotic Prophylaxis](https://www.webofscience.com/wos/alldb/full-record/WOS:000395646300018) |
|  | 1. [Impact of probiotics during weaning on the metabolic and inflammatory profile: follow-up at school age](https://www.webofscience.com/wos/alldb/full-record/WOS:000361335300012) |
|  | 1. [Agave fructans enhance the effects of fermented milk products on obesity biomarkers: a randomised trial](https://www.webofscience.com/wos/alldb/full-record/WOS:000971608000006) |
|  | 1. [Characterization of the Stool Microbiome in Hispanic Preschool Children by Weight Status and Time](https://www.webofscience.com/wos/alldb/full-record/WOS:000424406000007) |
|  | 1. [Effects of probiotics (Vivomixx (R)) in obese pregnant women and their newborn: study protocol for a randomized controlled trial](https://www.webofscience.com/wos/alldb/full-record/WOS:000384848600002) |
|  | 1. [The Effects of Limosilactobacillus reuteri LR-99 Supplementation on Body Mass Index, Social Communication, Fine Motor Function, and Gut Microbiome Composition in Individuals with Prader-Willi Syndrome: a Randomized Double-Blinded Placebo-Controlled Trial](https://www.webofscience.com/wos/alldb/full-record/WOS:000660484700001) |
|  | 1. [Effects of inulin supplementation on body composition and metabolic outcomes in children with obesity](https://www.webofscience.com/wos/alldb/full-record/WOS:000833335700015) |
|  | 1. [Effects of synbiotic supplementation on anthropometric indices and body composition in overweight or obese children and adolescents: a randomized, double-blind, placebo-controlled clinical trial](https://www.webofscience.com/wos/alldb/full-record/WOS:000896367600001) |
|  | 1. [Growth of infants consuming whey -predominant term infant formulas with a protein content of 1.8 g/100 kcal: a multicenter pooled analysis of individual participant data](https://www.webofscience.com/wos/alldb/full-record/WOS:000384874900019) |
|  | 1. [A randomized triple-masked controlled trial on the effects of synbiotics on inflammation markers in overweight children](https://www.webofscience.com/wos/alldb/full-record/WOS:000334016100009) |
|  | 1. [SPRING: an RCT study of probiotics in the prevention of gestational diabetes mellitus in overweight and obese women](https://www.webofscience.com/wos/alldb/full-record/WOS:000315802200001) |
|  | 1. [Human Milk Oligosaccharides Modulate Fecal Microbiota and Are Safe for Use in Children With Overweight: A Randomized Controlled Trial](https://www.webofscience.com/wos/alldb/full-record/WOS:000683060000029) |
|  | 1. [Effects of synbiotic on anthropometry, lipid profile and oxidative stress in obese children](https://www.webofscience.com/wos/alldb/full-record/WOS:000367307500002) |
|  | 1. [Probiotics during weaning: a follow-up study on effects on body composition and metabolic markers at school age](https://www.webofscience.com/wos/alldb/full-record/WOS:000351513000003) |
|  | 1. [Effects of Fecal Microbiome Transfer in Adolescents With Obesity The Gut Bugs Randomized Controlled Trial](https://www.webofscience.com/wos/alldb/full-record/WOS:000603058300007) |
|  | 1. [Oral administration of maternal vaginal microbes at birth to restore gut microbiome development in infants born by caesarean section: A pilot randomised placebo-controlled trial](https://www.webofscience.com/wos/alldb/full-record/WOS:000684979000019) |
|  | 1. [Effects of microbiota-directed foods in gnotobiotic animals and undernourished children](https://www.webofscience.com/wos/alldb/full-record/WOS:000474905400034) |
|  | 1. [Therapeutic Effects of Butyrate on Pediatric Obesity A Randomized Clinical Trial](https://www.webofscience.com/wos/alldb/full-record/WOS:000937072600006) |
|  | 1. [Probiotics for the Prevention of Gestational Diabetes Mellitus in Overweight and Obese Women: Findings From the SPRING Double-Blind Randomized Controlled Trial](https://www.webofscience.com/wos/alldb/full-record/WOS:000459181200015) |
|  | 1. [Effect of Lactobacillus salivarius Ls-33 on fecal microbiota in obese adolescents](https://www.webofscience.com/wos/alldb/full-record/WOS:000327911200008) |
|  | 1. [Reinforcement of intestinal epithelial barrier by arabinoxylans in overweight and obese subjects: A randomized controlled trial Arabinoxylans in gut barrier](https://www.webofscience.com/wos/alldb/full-record/WOS:000428483200007) |
|  | 1. [Long-term safety and efficacy of perinatal probiotic intervention: Evidence from a follow-up study of four randomized, double-blind, placebo-controlled trials](https://www.webofscience.com/wos/alldb/full-record/WOS:000394983000008) |
|  | 1. [Effects of Bifidobacterium animalis Subsp. lactis (BPL1) Supplementation in Children and Adolescents with Prader-Willi Syndrome: A Randomized Crossover Trial](https://www.webofscience.com/wos/alldb/full-record/WOS:000585280600001) |
|  | 1. [Effect of oligofructose supplementation on body weight in overweight and obese children: a randomised, double-blind, placebo-controlled trial](https://www.webofscience.com/wos/alldb/full-record/WOS:000347103800019) |
|  | 1. [Facilitating a high-quality dietary pattern induces shared microbial responses linking diet quality, blood pressure, and microbial sterol metabolism in caregiver-child dyads](https://www.webofscience.com/wos/alldb/full-record/WOS:000893739800001) |
|  | 1. [Fish Oil And/Or Probiotics Intervention in Overweight/Obese Pregnant Women and Overweight Risk in 24-Month-Old Children](https://www.webofscience.com/wos/alldb/full-record/WOS:000932048700022) |
|  | 1. [Prebiotics Reduce Body Fat and Alter Intestinal Microbiota in Children Who Are Overweight or With Obesity](https://www.webofscience.com/wos/alldb/full-record/WOS:000408703800027) |
|  | 1. [Metagenomics analysis of gut microbiota in response to diet intervention and gestational diabetes in overweight and obese women: a randomised, double-blind, placebo-controlled clinical trial](https://www.webofscience.com/wos/alldb/full-record/WOS:000609237100012) |
|  | 1. [Prebiotic effect on mood in obese patients is determined by the initial gut microbiota composition: A randomized, controlled trial](https://www.webofscience.com/wos/alldb/full-record/WOS:000640700200007) |
|  | 1. [A Vegetarian Diet Is a Major Determinant of Gut Microbiota Composition in Early Pregnancy](https://www.webofscience.com/wos/alldb/full-record/WOS:000447544900093) |
|  | 1. [Evaluation of the effect of Lactobacillus reuteri V3401 on biomarkers of inflammation, cardiovascular risk and liver steatosis in obese adults with metabolic syndrome: a randomized clinical trial (PROSIR)](https://www.webofscience.com/wos/alldb/full-record/WOS:000451076900001) |
|  | 1. [Link between gut microbiota and health outcomes in inulin -treated obese patients: Lessons from the Food4Gut multicenter randomized placebo-controlled trial](https://www.webofscience.com/wos/alldb/full-record/WOS:000600555900007) |
|  | 1. [Probiotic therapy outcomes in body composition of children and adolescent with obesity, a nonrandomized controlled trial](https://www.webofscience.com/wos/alldb/full-record/WOS:000891248000005) |
|  | 1. [Effects of Probiotics on Nonalcoholic Fatty Liver Disease in Obese Children and Adolescents](https://www.webofscience.com/wos/alldb/full-record/WOS:000395525800028) |
|  | 1. [Tenth year reenrollment randomized trial investigating the effects of childhood probiotics and calcium supplementation on height and weight at adolescence](https://www.webofscience.com/wos/alldb/full-record/WOS:000687328100010) |
|  | 1. [Strain engraftment competition and functional augmentation in a multi-donor fecal microbiota transplantation trial for obesity](https://www.webofscience.com/wos/alldb/full-record/WOS:000650062900001) |
|  | 1. [Effect of Mastiha supplementation on NAFLD: The MAST4HEALTH Randomised, Controlled Trial](https://www.webofscience.com/wos/alldb/full-record/WOS:000640658800001) |
|  | 1. [Effect of Synbiotic Supplementation in a Very-Low-Calorie Ketogenic Diet on Weight Loss Achievement and Gut Microbiota: A Randomized Controlled Pilot Study](https://www.webofscience.com/wos/alldb/full-record/WOS:000484197700001) |
|  | 1. [Consuming yellow pea fiber reduces voluntary energy intake and body fat in overweight/obese adults in a 12-week randomized controlled trial](https://www.webofscience.com/wos/alldb/full-record/WOS:000397833800012) |
|  | 1. [Study protocol: optimized complementary feeding study (OTIS): a randomized controlled trial of the impact of a protein-reduced complementary diet based on Nordic foods](https://www.webofscience.com/wos/alldb/full-record/WOS:000457471800007) |
|  | 1. [Nutrition program, physical activity and gut microbiota modulation: a randomized controlled trial to promote a healthy lifestyle in students with vitamin D-3 deficiency](https://www.webofscience.com/wos/alldb/full-record/WOS:000836821100010) |
|  | 1. [Improved Plasma Lipids, Anti-Inflammatory Activity, and Microbiome Shifts in Overweight Participants: Two Clinical Studies on Oral Supplementation with Algal Sulfated Polysaccharide](https://www.webofscience.com/wos/alldb/full-record/WOS:000845747200001) |
|  | 1. [Arabinoxylan oligosaccharides and polyunsaturated fatty acid effects on gut microbiota and metabolic markers in overweight individuals with signs of metabolic syndrome: A randomized cross-over trial](https://www.webofscience.com/wos/alldb/full-record/WOS:000510526200008) |
|  | 1. [Probiotics to Adolescents With Obesity: Effects on Inflammation and Metabolic Syndrome](https://www.webofscience.com/wos/alldb/full-record/WOS:000311702500017) |
|  | 1. [Effect of prebiotic intake on gut microbiota, intestinal permeability and glycemic control in children with type 1 diabetes: study protocol for a randomized controlled trial](https://www.webofscience.com/wos/alldb/full-record/WOS:000380104900001) |
|  | 1. [Impact of maternal probiotic-supplemented dietary counselling on pregnancy outcome and prenatal and postnatal growth: a double-blind, placebo-controlled study](https://www.webofscience.com/wos/alldb/full-record/WOS:000279292200014) |
|  | 1. [Impact of dietary fiber supplementation on modulating microbiota-host-metabolic axes in obesity](https://www.webofscience.com/wos/alldb/full-record/WOS:000459081600023) |
|  | 1. [Nutrient Intake and Gut Microbial Genera Changes after a 4-Week Placebo Controlled Galacto-Oligosaccharides Intervention in Young Females](https://www.webofscience.com/wos/alldb/full-record/WOS:000736902200001) |
|  | 1. [Rifaximin alters gut microbiota profile, but does not affect systemic inflammation - a randomized controlled trial in common variable immunodeficiency](https://www.webofscience.com/wos/alldb/full-record/WOS:000455951300015) |
|  | 1. [Randomised clinical trial: the beneficial effects of VSL# 3 in obese children with non-alcoholic steatohepatitis](https://www.webofscience.com/wos/alldb/full-record/WOS:000335371000003) |
|  | 1. [Effects of Lactobacillus rhamnosus Strain GG in Pediatric Obesity-related Liver Disease](https://www.webofscience.com/wos/alldb/full-record/WOS:000290750500016) |
|  | 1. [A maternal higher-complex carbohydrate diet increases bifidobacteria and alters early life acquisition of the infant microbiome in women with gestational diabetes mellitus](https://www.webofscience.com/wos/alldb/full-record/WOS:000839006700001) |
|  | 1. [Impact of probiotic feeding during weaning on the serum lipid profile and plasma metabolome in infants](https://www.webofscience.com/wos/alldb/full-record/WOS:000320123500013) |
|  | 1. [Probiotics and Maternal Mental Health: A Randomised Controlled Trial among Pregnant Women with Obesity](https://www.webofscience.com/wos/alldb/full-record/WOS:000562860600014) |
|  | 1. [An alcohol-free beer enriched with isomaltulose and a resistant dextrin modulates gut microbiome in subjects with type 2 diabetes mellitus and overweight or obesity: a pilot study](https://www.webofscience.com/wos/alldb/full-record/WOS:000635212200001) |
|  | 1. [The Effect of Weaning with Adult Food Typical of the Mediterranean Diet on Taste Development and Eating Habits of Children: A Randomized Trial](https://www.webofscience.com/wos/alldb/full-record/WOS:000817501300001) |
|  | 1. [Gut microbiota manipulation with prebiotics in patients with non-alcoholic fatty liver disease: a randomized controlled trial protocol](https://www.webofscience.com/wos/alldb/full-record/WOS:000365942800001) |
|  | 1. [PHAGE Study: Effects of Supplemental Bacteriophage Intake on Inflammation and Gut Microbiota in Healthy Adults](https://www.webofscience.com/wos/alldb/full-record/WOS:000464367000006) |
|  | 1. [Targeting the Intestinal Microbiota to Prevent Type 2 Diabetes and Enhance the Effect of Metformin on Glycaemia: A Randomised Controlled Pilot Study](https://www.webofscience.com/wos/alldb/full-record/WOS:000554638700001) |
|  | 1. [Late preterm birth has direct and indirect effects on infant gut microbiota development during the first six months of life](https://www.webofscience.com/wos/alldb/full-record/WOS:000405216700023) |
|  | 1. [Yogurt consumption during pregnancy and preterm delivery in Mexican women: A prospective analysis of interaction with maternal overweight status](https://www.webofscience.com/wos/alldb/full-record/WOS:000428452800009) |
|  | 1. [Impact of probiotics supplement on the gut microbiota in neonates with antibiotic exposure: an open-label single-center randomized parallel controlled study](https://www.webofscience.com/wos/alldb/full-record/WOS:000679779200001) |
|  | 1. [Evaluation of yellow pea fibre supplementation on weight loss and the gut microbiota: a randomized controlled trial](https://www.webofscience.com/wos/alldb/full-record/WOS:000334421200002) |
|  | 1. [Impact of wheat aleurone on biomarkers of cardiovascular disease, gut microbiota and metabolites in adults with high body mass index: a double-blind, placebo-controlled, randomized clinical trial](https://www.webofscience.com/wos/alldb/full-record/WOS:000764906600002) |
|  | 1. [Microbiota engraftment after faecal microbiota transplantation in obese subjects with type 2 diabetes: a 24-week, double-blind, randomised controlled trial](https://www.webofscience.com/wos/alldb/full-record/WOS:000728868500001) |
|  | 1. [The impact of probiotic supplementation during pregnancy on DNA methylation of obesity-related genes in mothers and their children](https://www.webofscience.com/wos/alldb/full-record/WOS:000461781300031) |
|  | 1. [Probiotics reduce self-reported symptoms of upper respiratory tract infection in overweight and obese adults: should we be considering probiotics during viral pandemics? (vol 13, 1900997, 2021)](https://www.webofscience.com/wos/alldb/full-record/WOS:000709845500001) |
|  | 1. [Structural Alteration of Gut Microbiota during the Amelioration of Human Type 2 Diabetes with Hyperlipidemia by Metformin and a Traditional Chinese Herbal Formula: a Multicenter, Randomized, Open Label Clinical Trial](https://www.webofscience.com/wos/alldb/full-record/WOS:000433051200019) |
|  | 1. [The effect of synbiotic supplementation on anthropometric indices, appetite, and constipation in people with hypothyroidism: A randomized, double-blind, placebo-controlled trial](https://www.webofscience.com/wos/alldb/full-record/WOS:000529798500001) |
|  | 1. [Metabolic phenotypes and the gut microbiota in response to dietary resistant starch type 2 in normal-weight subjects: a randomized crossover trial](https://www.webofscience.com/wos/alldb/full-record/WOS:000461761200001) |
|  | 1. [Association of human milk oligosaccharides and nutritional status of young infants among Bangladeshi mother-infant dyads](https://www.webofscience.com/wos/alldb/full-record/WOS:000809083500054) |
|  | 1. [Altered Salivary Microbiota Following Bifidobacterium animalis Subsp. Lactis BL-11 Supplementation Are Associated with Anthropometric Growth and Social Behavior Severity in Individuals with Prader-Willi Syndrome](https://www.webofscience.com/wos/alldb/full-record/WOS:000787637600001) |
|  | 1. [Disrupted Intestinal Microbiota and Intestinal Inflammation in Children with Cystic Fibrosis and Its Restoration with Lactobacillus GG: A Randomised Clinical Trial](https://www.webofscience.com/wos/alldb/full-record/WOS:000331711900018) |
|  | 1. [A specific dietary fibre supplementation improves cognitive performance-an exploratory randomised, placebo-controlled, crossover study](https://www.webofscience.com/wos/alldb/full-record/WOS:000571037400001) |
|  | 1. [A Mixture of trans-Galactooligosaccharides Reduces Markers of Metabolic Syndrome and Modulates the Fecal Microbiota and Immune Function of Overweight Adults](https://www.webofscience.com/wos/alldb/full-record/WOS:000315173500011) |
|  | 1. [The Commensal Microbe Veillonella as a Marker for Response to an FGF19 Analog in NASH](https://www.webofscience.com/wos/alldb/full-record/WOS:000597255900001) |
|  | 1. [Effects of Regular Kefir Consumption on Gut Microbiota in Patients with Metabolic Syndrome: A Parallel-Group, Randomized, Controlled Study](https://www.webofscience.com/wos/alldb/full-record/WOS:000487964600296) |
|  | 1. [Enhanced nutrient supply and intestinal microbiota development in very low birth weight infants](https://www.webofscience.com/wos/alldb/full-record/WOS:000481648100009) |
|  | 1. [Effect of probiotic yoghurt on plasma glucose in overweight and obese pregnant women: a randomized controlled clinical trial](https://www.webofscience.com/wos/alldb/full-record/WOS:000512078900019) |
|  | 1. [The Effect of Lean-Seafood and Non-Seafood Diets on Fecal Metabolites and Gut Microbiome: Results from a Randomized Crossover Intervention Study](https://www.webofscience.com/wos/alldb/full-record/WOS:000454686700008) |
|  | 1. [Effects of underfeeding and oral vancomycin on gut microbiome and nutrient absorption in humans](https://www.webofscience.com/wos/alldb/full-record/WOS:000521529700003) |
|  | 1. [A placebo-controlled trial of Lactobacillus GG to prevent diarrhea in undernourished Peruvian children](https://www.webofscience.com/wos/alldb/full-record/MEDLINE:9880443) |
|  | 1. [Inulin-type fructans modulate intestinal Bifidobacterium species populations and decrease fecal short-chain fatty acids in obese women](https://www.webofscience.com/wos/alldb/full-record/WOS:000355034900024) |
|  | 1. [Fructooligosaccharide (FOS) and Galactooligosaccharide (GOS) Increase Bifidobacterium but Reduce Butyrate Producing Bacteria with Adverse Glycemic Metabolism in healthy young population](https://www.webofscience.com/wos/alldb/full-record/WOS:000410916800003) |
|  | 1. [Characterizing the Effects of Calcium and Prebiotic Fiber on Human Gut Microbiota Composition and Function Using a Randomized Crossover Design-A Feasibility Study](https://www.webofscience.com/wos/alldb/full-record/WOS:000666661600001) |
|  | 1. [Metformin alters the gut microbiome of individuals with treatment-naive type 2 diabetes, contributing to the therapeutic effects of the drug](https://www.webofscience.com/wos/alldb/full-record/WOS:000405180100014) |
|  | 1. [Effect of antenatal dietary interventions in maternal obesity on pregnancy weight-gain and birthweight: Healthy Mums and Babies (HUMBA) randomized trial](https://www.webofscience.com/wos/alldb/full-record/WOS:000477565500021) |
|  | 1. [Impact of dietary counselling and probiotic intervention on maternal anthropometric measurements during and after pregnancy: A randomized placebo-controlled trial](https://www.webofscience.com/wos/alldb/full-record/WOS:000290009800004) |
|  | 1. [Investigation the effect of oleoylethanolamide supplementation on the abundance of Akkermansia muciniphila bacterium and the dietary intakes in people with obesity: A randomized clinical trial](https://www.webofscience.com/wos/alldb/full-record/WOS:000477690300010) |
|  | 1. The Effect of Synbiotic Supplementation on Growth Parameters in Mild to Moderate FTT Children Aged 2-5 Years |
|  | 1. [The Effects of Probiotic Supplements on Blood Markers of Endotoxin and Lipid Peroxidation in Patients Undergoing Gastric Bypass Surgery; a Randomized, Double-Blind, Placebo-Controlled, Clinical Trial with 13 Months Follow-Up](https://www.webofscience.com/wos/alldb/full-record/WOS:000467148300025) |
|  | 1. [Mixed Spices at Culinary Doses Have Prebiotic Effects in Healthy Adults: A Pilot Study](https://www.webofscience.com/wos/alldb/full-record/WOS:000474936700231) |
|  | 1. [Effects of probiotics in patients with diabetes mellitus type 2: study protocol for a randomized, double-blind, placebo-controlled trial](https://www.webofscience.com/wos/alldb/full-record/WOS:000321584300001) |
|  | 1. [Is there a value for probiotic supplements in gestational diabetes mellitus? A randomized clinical trial](https://www.webofscience.com/wos/alldb/full-record/WOS:000411107500001) |
|  | 1. [The effects of prebiotics on gastrointestinal side effects of metformin in youth: A pilot randomized control trial in youth-onset type 2 diabetes](https://www.webofscience.com/wos/alldb/full-record/WOS:000946336300001) |
|  | 1. [Cardiovascular Benefits of Empagliflozin Are Associated With Gut Microbiota and Plasma Metabolites in Type 2 Diabetes](https://www.webofscience.com/wos/alldb/full-record/WOS:000784953300001) |
|  | 1. [Nutritional Intervention Preconception and During Pregnancy to Maintain Healthy Glucose Metabolism and Offspring Health ("NiPPeR"): study protocol for a randomised controlled trial](https://www.webofscience.com/wos/alldb/full-record/WOS:000397000600001) |
|  | 1. [Microbial enterotypes beyond genus level: Bacteroides species as a predictive biomarker for weight change upon controlled intervention with arabinoxylan oligosaccharides in overweight subjects](https://www.webofscience.com/wos/alldb/full-record/WOS:000598606200001) |
|  | 1. [Supplementation of Diet With Galacto-oligosaccharides Increases Bifidobacteria, but Not Insulin Sensitivity, in Obese Prediabetic Individuals](https://www.webofscience.com/wos/alldb/full-record/WOS:000403918300027) |
|  | 1. [Effect of probiotic Lactobacillus plantarum Dad-13 powder consumption on the gut microbiota and intestinal health of overweight adults](https://www.webofscience.com/wos/alldb/full-record/WOS:000609418200008) |
|  | 1. [A randomised controlled demonstration trial of multifaceted nutritional intervention and or probiotics: the healthy mums and babies (HUMBA) trial](https://www.webofscience.com/wos/alldb/full-record/WOS:000442406600002) |
|  | 1. [Effect of alive probiotic on insulin resistance in type 2 diabetes patients: Randomized clinical trial](https://www.webofscience.com/wos/alldb/full-record/MEDLINE:29661605) |
|  | 1. [The Effects of Synbiotic Supplementation on Carotid Intima-Media Thickness, Biomarkers of Inflammation, and Oxidative Stress in People with Overweight, Diabetes, and Coronary Heart Disease: a Randomized, Double-Blind, Placebo-Controlled Trial](https://www.webofscience.com/wos/alldb/full-record/WOS:000471045000014) |
|  | 1. [Effects of a Diet-Based Weight-Reducing Program with Probiotic Supplementation on Satiety Efficiency, Eating Behaviour Traits, and Psychosocial Behaviours in Obese Individuals](https://www.webofscience.com/wos/alldb/full-record/WOS:000397023600103) |
|  | 1. [Effects of short-term supplementation with bovine lactoferrin and/or immunoglobulins on body mass and metabolic measures: a randomised controlled trial](https://www.webofscience.com/wos/alldb/full-record/WOS:000394029400010) |
|  | 1. [The acute effects of inulin and resistant starch on postprandial serum short-chain fatty acids and second-meal glycemic response in lean and overweight humans](https://www.webofscience.com/wos/alldb/full-record/WOS:000394027500014) |
|  | 1. [The effects of fermented vegetable consumption on the composition of the intestinal microbiota and levels of inflammatory markers in women: A pilot and feasibility study](https://www.webofscience.com/wos/alldb/full-record/WOS:000925060900060) |
|  | 1. [Effects of long-term weekly iron and folic acid supplementation on lower genital tract infection - a double blind, randomised controlled trial in Burkina Faso](https://www.webofscience.com/wos/alldb/full-record/WOS:000416043900001) |
|  | 1. [Insight into the prebiotic concept: lessons from an exploratory, double blind intervention study with inulin-type fructans in obese women](https://www.webofscience.com/wos/alldb/full-record/WOS:000321337600006) |
|  | 1. [Safety and tolerability of experimental hookworm infection in humans with metabolic disease: study protocol for a phase 1b randomised controlled clinical trial](https://www.webofscience.com/wos/alldb/full-record/WOS:000511655800001) |
|  | 1. Duodenal Anaerobutyricum soehngenii infusion stimulates GLP-1 production, ameliorates glycaemic control and beneficially shapes the duodenal transcriptome in metabolic syndrome subjects: a randomised double-blind placebo-controlled cross-over study |
|  | 1. [Impact of probiotics in women with gestational diabetes mellitus on metabolic health: a randomized controlled trial](https://www.webofscience.com/wos/alldb/full-record/WOS:000352147100021) |
|  | 1. [A randomised controlled trial of a duodenal-jejunal bypass sleeve device (EndoBarrier) compared with standard medical therapy for the management of obese subjects with type 2 diabetes mellitus](https://www.webofscience.com/wos/alldb/full-record/WOS:000422898800225) |
|  | 1. [Effect of probiotics on postoperative quality of gastric bypass surgeries: a prospective randomized trial](https://www.webofscience.com/wos/alldb/full-record/WOS:000369466900008) |
|  | 1. [Effect of an infant formula containing sn-2 palmitate on fecal microbiota and metabolome profiles of healthy term infants: a randomized, double-blind, parallel, controlled study](https://www.webofscience.com/wos/alldb/full-record/WOS:000748632900001) |
|  | 1. [Effects of Multistrain Probiotic Supplementation on Glycemic and Inflammatory Indices in Patients with Nonalcoholic Fatty Liver Disease: A Double-Blind Randomized Clinical Trial](https://www.webofscience.com/wos/alldb/full-record/WOS:000382977400002) |
|  | 1. [Effects of synbiotic food consumption on glycemic status and serum hs-CRP in pregnant women: a randomized controlled clinical trial](https://www.webofscience.com/wos/alldb/full-record/WOS:000342611200012) |
|  | 1. [Effect of Lactobacillus rhamnosus CGMCC1.3724 supplementation on weight loss and maintenance in obese men and women](https://www.webofscience.com/wos/alldb/full-record/WOS:000333559700018) |
|  | 1. [The effects of dietary supplementation with inulin and inulin-propionate ester on hepatic steatosis in adults with non-alcoholic fatty liver disease](https://www.webofscience.com/wos/alldb/full-record/WOS:000455806600021) |
|  | 1. [The rationale and design of the personal diet study, a randomized clinical trial evaluating a personalized approach to weight loss in individuals with pre-diabetes and early-stage type 2 diabetes](https://www.webofscience.com/wos/alldb/full-record/WOS:000463982400010) |
|  | 1. [Probiotics and prebiotics for severe acute malnutrition (PRONUT study): a double-blind efficacy randomised controlled trial in Malawi](https://www.webofscience.com/wos/alldb/full-record/WOS:000268111600031) |
|  | 1. [Weight and Glucose Reduction Observed with a Combination of Nutritional Agents in Rodent Models Does Not Translate to Humans in a Randomized Clinical Trial with Healthy Volunteers and Subjects with Type 2 Diabetes](https://www.webofscience.com/wos/alldb/full-record/WOS:000374541200014) |
|  | 1. [Effect of the intake of dietary protein on insulin resistance in subjects with obesity: a randomized controlled clinical trial](https://www.webofscience.com/wos/alldb/full-record/WOS:000585034400001) |
|  | 1. [Effects of plant stanol ester consumption on fasting plasma oxy(phyto) sterol concentrations as related to fecal microbiota characteristics](https://www.webofscience.com/wos/alldb/full-record/WOS:000401391300007) |
|  | 1. [Acute increases in serum colonic short-chain fatty acids elicited by inulin do not increase GLP-1 or PYY responses but may reduce ghrelin in lean and overweight humans](https://www.webofscience.com/wos/alldb/full-record/WOS:000406963700005) |
|  | 1. [The efficacy of probiotics and/or n-3 long-chain polyunsaturated fatty acids intervention on maternal prenatal and postnatal depressive and anxiety symptoms among overweight and obese women](https://www.webofscience.com/wos/alldb/full-record/WOS:000656574100004) |
|  | 1. [alpha-Galacto-oligosaccharides Dose-Dependently Reduce Appetite and Decrease Inflammation in Overweight Adults](https://www.webofscience.com/wos/alldb/full-record/WOS:000360915100011) |
|  | 1. [Effect of maternal supplement beverage with and without probiotics during pregnancy and lactation on maternal and infant health: a randomized controlled trial in the Philippines](https://www.webofscience.com/wos/alldb/full-record/WOS:000434292100001) |
|  | 1. [Feasibility Study of Lactobacillus Plantarum 299v Probiotic Supplementation in an Urban Academic Facility among Diverse Pregnant Individuals](https://www.webofscience.com/wos/alldb/full-record/WOS:000939954100001) |
|  | 1. [Effect of synbiotic pomegranate juice on glycemic, sex hormone profile and anthropometric indices in PCOS: A randomized, triple blind, controlled trial](https://www.webofscience.com/wos/alldb/full-record/WOS:000456727900012) |
|  | 1. [Benefits of repeated individual dietary counselling in long-term weight control in women after delivery](https://www.webofscience.com/wos/alldb/full-record/WOS:000362197100050) |
|  | 1. [Short-term impact of sucralose consumption on the metabolic response and gut microbiome of healthy adults](https://www.webofscience.com/wos/alldb/full-record/WOS:000506231000002) |
|  | 1. [Effects of Probiotic Supplementation on Hormonal Profiles, Biomarkers of Inflammation and Oxidative Stress in Women With Polycystic Ovary Syndrome: A Randomized, Double-Blind, Placebo-Controlled Trial](https://www.webofscience.com/wos/alldb/full-record/WOS:000428184200001) |
|  | 1. [Comparison of the effect of daily consumption of probiotic compared with low-fat conventional yogurt on weight loss in healthy obese women following an energy-restricted diet: a randomized controlled trial](https://www.webofscience.com/wos/alldb/full-record/WOS:000369465400006) |
|  | 1. [Probiotics Lactobacillus reuteri DSM 17938 and Lactobacillus casei CRL 431 Modestly Increase Growth, but Not Iron and Zinc Status, among Indonesian Children Aged 1-6 Years](https://www.webofscience.com/wos/alldb/full-record/WOS:000320894800024) |
|  | 1. [Daily Inclusion of Resistant Starch-Containing Potatoes in a Dietary Guidelines for Americans Dietary Pattern Does Not Adversely Affect Cardiometabolic Risk or Intestinal Permeability in Adults with Metabolic Syndrome: A Randomized Controlled Trial](https://www.webofscience.com/wos/alldb/full-record/WOS:000786168400001) |
|  | 1. [Probiotic Supplementation Attenuates Increases in Body Mass and Fat Mass During High-Fat Diet in Healthy Young Adults](https://www.webofscience.com/wos/alldb/full-record/WOS:000367189300015) |
|  | 1. [The study protocol for a pseudo-randomised pre-post designed controlled intervention trial to study the effects of a 7-week cooking program on self-efficacy and biomarkers of health: the ECU lifestyle and biomarkers get connected study (ECULABJMOF) including the Jamie's Ministry of Food WA participant experience](https://www.webofscience.com/wos/alldb/full-record/WOS:000546835600002) |
|  | 1. [Probiotic Supplementation and Trimethylamine-N-Oxide Production Following a High-Fat Diet](https://www.webofscience.com/wos/alldb/full-record/WOS:000367189300014) |
|  | 1. [Inulin-enriched pasta improves intestinal permeability and modifies the circulating levels of zonulin and glucagon-like peptide 2 in healthy young volunteers](https://www.webofscience.com/wos/alldb/full-record/WOS:000314481600005) |
|  | 1. [Beneficial effects of Bifidobacterium lactis on lipid profile and cytokines in patients with metabolic syndrome: A randomized trial. Effects of probiotics on metabolic syndrome](https://www.webofscience.com/wos/alldb/full-record/WOS:000375371700016) |
|  | 1. [Eight weeks of lentil consumption attenuates insulin resistance progression without increased gastrointestinal symptom severity: A randomized clinical trial](https://www.webofscience.com/wos/alldb/full-record/WOS:000862510400002) |
|  | 1. [Human gut microbiome composition and tryptophan metabolites were changed differently by fast food and Mediterranean diet in 4 days: a pilot study](https://www.webofscience.com/wos/alldb/full-record/WOS:000548521300006) |
|  | 1. [Effects of synbiotic supplementation on insulin resistance in subjects with the metabolic syndrome: a randomised, double-blind, placebo-controlled pilot study](https://www.webofscience.com/wos/alldb/full-record/WOS:000339054100016) |
|  | 1. [Effect of daily consumption of probiotic yoghurt on insulin resistance in pregnant women: a randomized controlled trial](https://www.webofscience.com/wos/alldb/full-record/WOS:000313527300014) |
|  | 1. [Lower Pretreatment Gut Integrity Is Independently Associated With Fat Gain on Antiretroviral Therapy](https://www.webofscience.com/wos/alldb/full-record/WOS:000464937800020) |
|  | 1. [Effects of whole-grain rye porridge with added inulin and wheat gluten on appetite, gut fermentation and postprandial glucose metabolism: a randomised, cross-over, breakfast study](https://www.webofscience.com/wos/alldb/full-record/WOS:000392726300015) |
| SinoMed  （5） | 1. Can probiotic supplementation improve and control appetite in overweight and obese children |
|  | 1. Effect of vitamin d combined with Saccharomyces boulardii on the intervention of obese children |
|  | 1. Effect of probiotics on nonalcoholic fatty liver in obese children and adolescents |
|  | 1. Alleviation of dss-induced ulcerative colitis in mice by a human strain of Bifidobacterium pseudobulbarum |
|  | 1. Survey on the changes of intestinal flora and dietary structure of obese children aged 3-6 years in urban Chengdu and analysis of factors influencing childhood obesity |
| CNKI  （4） | 1. Intervention study on energy metabolism and inflammatory factors in children with simple obesity by Lactobacillus bifidum triptans tablets |
|  | 1. Triple active probiotics improve intestinal dysbiosis and fat metabolism in overweight and obese children |
|  | 1. A randomized, double-blind, placebo-controlled clinical study of probiotic intervention in the conversion of abnormal glucose tolerance to type 2 diabetes |
|  | 1. Clinical observation on the effect of probiotics on leptin, lipocalin and blood lipid levels in children with simple obesity |

**Table S2.** Specific review annotations for remaining 317 articles after removing duplicates.

| Reviewed Papers（317） | Review Annotations | Reviewer |
| --- | --- | --- |
| 1. Can probiotic supplementation improve and control appetite in overweight and obese children | Exclusion for review article | YL/LW |
| 1. Intervention study on energy metabolism and inflammatory factors in children with simple obesity by Lactobacillus bifidum triplex tablets | Meeting the inclusion criteria for meta-analysis and systematic review | YL/LW |
| 1. Triple active probiotics improve intestinal dysbiosis and fat metabolism in overweight and obese children | Exclusion for no full text | YL/LW |
| 1. Effect of vitamin d combined with *Saccharomyces boulardii* on the intervention of obese children | Exclusion for not only with probiotic intervention | YL/LW |
| 1. Effect of probiotics on nonalcoholic fatty liver in obese children and adolescents | Exclusion for not overweight or obesity | YL/LW |
| 1. Alleviation of dss-induced ulcerative colitis in mice by a human strain of *Bifidobacterium pseudobulbarum* | Exclusion for animal experiment | YL/LW |
| 1. A randomized, double-blind, placebo-controlled clinical study of probiotic intervention in the conversion of abnormal glucose tolerance to type 2 diabetes | Exclusion for not overweight or obesity | YL/LW |
| 1. Clinical observation on the effect of probiotics on leptin, lipocalin and blood lipid levels in children with simple obesity | Meeting the inclusion criteria for meta-analysis and systematic review | YL/LW |
| 1. Survey on the changes of intestinal flora and dietary structure of obese children aged 3-6 years in urban Chengdu and analysis of factors influencing childhood obesity | Exclusion for review article | YL/LW |
| 1. Gut Bugs Trial - Gut microbiome transfer for the treatment of adolescent obesity | Exclusion for not probiotic intervention | YL/LW |
| 1. Vegetables as first foods for babies study | Exclusion for not children | YL/LW |
| 1. The Effect of Synbiotic Supplementation on Growth Parameters in Mild to Moderate FTT Children Aged 2-5 Years | Exclusion for not overweight or obesity | YL/LW |
| 1. Lifestyle Interventions Including Nutrition, Exercise, and Supplements for Nonalcoholic Fatty Liver Disease in Children | Exclusion for not overweight or obesity | YL/LW |
| 1. Hydrolyzed Formula With Reduced Protein Content Supports Adequate Growth: a Randomized Controlled Noninferiority Trial | Exclusion for not children | YL/LW |
| 1. Growth of infants consuming whey -predominant term infant formulas with a protein content of 1.8 g/100 kcal: a multicenter pooled analysis of individual participant data | Exclusion for not children | YL/LW |
| 1. Randomised clinical trial: the beneficial effects of VSL# 3 in obese children with non-alcoholic steatohepatitis | Exclusion for not overweight or obesity | YL/LW |
| 1. Effects of probiotics in patients with diabetes mellitus type 2: study protocol for a randomized, double-blind, placebo-controlled trial | Exclusion for study protocol | YL/LW |
| 1. Effects of Bifidobacterium animalis Subsp. lactis (BPL1) Supplementation in Children and Adolescents with Prader-Willi Syndrome: A Randomized Crossover Trial | Exclusion for not overweight or obesity | YL/LW |
| 1. Nutrition Society of New Zealand Annual Conference | Exclusion for review article | YL/LW |
| 1. Probiotics and Pregnancy | Exclusion for review article | YL/LW |
| 1. Rationale and design of "Hearts & Parks": study protocol for a pragmatic randomized clinical trial of an integrated clinic-community intervention to treat pediatric obesity | Exclusion for study protocol | YL/LW |
| 1. Effect of probiotic yoghurt on plasma glucose in overweight and obese pregnant women: a randomized controlled clinical trial | Exclusion for not children | YL/LW |
| 1. Effects of synbiotic supplementation on anthropometric indices and body composition in overweight or obese children and adolescents: a randomized, double-blind, placebo-controlled clinical trial | Exclusion for not probiotic intervention | YL/LW |
| 1. Beneficial Effects of Anti-Inflammatory Diet in Modulating Gut Microbiota and Controlling Obesity | Exclusion for not probiotic intervention | YL/LW |
| 1. A Vegetarian Diet Is a Major Determinant of Gut Microbiota Composition in Early Pregnancy | Exclusion for not probiotic intervention | YL/LW |
| 1. Probiotics for preventing gestational diabetes | Exclusion for not overweight or obesity | YL/LW |
| 1. Effects of underfeeding and oral vancomycin on gut microbiome and nutrient absorption in humans | Exclusion for not probiotic intervention | YL/LW |
| 1. The usefulness of probiotic administration in obese children with nonalcoholic fatty liver disease | Exclusion for obesity with other disease | YL/LW |
| 1. Beneficial effects of *Bifidobacterium lactis* on lipid profile and cytokines in patients with metabolic syndrome: a randomized trial. Effects of probiotics on metabolic syndrome | Exclusion for not children | YL/LW |
| 1. Exploring the Impact of Child-Centered Play Therapy on Academic Achievement of At-Risk Kindergarten Students | Exclusion for not overweight or obesity | YL/LW |
| 1. Study Protocol of a Multicenter Randomized Controlled Trial to Tackle Obesity through a Mediterranean Diet vs. a Traditional Low-Fat Diet in Adolescents: The MED4Youth Study | Exclusion for study protocol | YL/LW |
| 1. Probiotic supplementation and trimethylamine-N-oxide production following a high-fat diet | Exclusion for not children | YL/LW |
| 1. Therapeutic microbiology: the role of bifidobacterium breve as food supplement for the prevention/treatment of paediatric diseases | Exclusion for review article | YL/LW |
| 1. Diet, Stress and Mental Health | Exclusion for review article | YL/LW |
| 1. Paradigm Shift in Allergy Prevention | Exclusion for not overweight or obesity | YL/LW |
| 1. Disrupted Intestinal Microbiota and Intestinal Inflammation in Children with Cystic Fibrosis and Its Restoration with Lactobacillus GG: A Randomised Clinical Trial | Exclusion for not overweight or obesity | YL/LW |
| 1. Maternal bacteria to correct abnormal gut microbiota in babies born by C-section | Exclusion for not probiotic intervention | YL/LW |
| 1. Benefits of Physical Exercise as Approach to Prevention and Reversion of Non-Alcoholic Fatty Liver Disease in Children and Adolescents with Obesity | Exclusion for not probiotic intervention | YL/LW |
| 1. Probiotics for the Prevention of Gestational Diabetes Mellitus in Overweight and Obese Women: Findings From the SPRING Double-Blind Randomized Controlled Trial | Exclusion for not children | YL/LW |
| 1. Complementary Feeding in Developed Countries: The 3 Ws (When, What, and Why?) | Exclusion for review article | YL/LW |
| 1. Effects of Dietary Iron Modulation on Gut Microbial Composition and Function in Monogastrics: A Review | Exclusion for review article | YL/LW |
| 1. A multi-strain probiotic blend reshaped obesity-related gut dysbiosis and improved lipid metabolism in obese children | Meeting the inclusion criteria for meta-analysis and systematic review | YL/LW |
| 1. Effect of probiotics on postoperative quality of gastric bypass surgeries: a prospective randomized trial | Exclusion for not overweight or obesity | YL/LW |
| 1. Effect of probiotics intake on obese children | Exclusion for no full text | YL/LW |
| 1. Probiotic mixture VSL#3: An overview of basic and clinical studies in chronic diseases | Exclusion for review article | YL/LW |
| 1. A synbiotic mixture of scGOS/lcFOS and Bifidobacterium breve M-16V is able to restore the delayed colonization of bifidobacterium observed in C-section Delivered Infants | Exclusion for not children | YL/LW |
| 1. Human Milk Oligosaccharide Supplementation Affects Intestinal Barrier Function and Microbial Composition in the Gastrointestinal Tract of Young Sprague Dawley Rats | Exclusion for animal experiment | YL/LW |
| 1. Effect of pre/probiotic mixture in children with nonalcoholic fatty liver disease: a randomized controlled trial | Exclusion for not overweight or obesity | YL/LW |
| 1. Impact of probiotic feeding during weaning on the serum lipid profile and plasma metabolome in infants | Exclusion for not children | YL/LW |
| 1. Microbial enterotypes beyond genus level: bacteroides species as a predictive biomarker for weight change upon controlled intervention with arabinoxylan oligosaccharides in overweight subjects | Exclusion for not children | YL/LW |
| 1. Intermittent fasting in experimental autoimmune encephalomyelitis and multiple sclerosis | Exclusion for not probiotic intervention | YL/LW |
| 1. Therapeutic Microbiology: The Role of Bifidobacterium breve as Food Supplement for the Prevention/Treatment of Paediatric Diseases | Exclusion for review article | YL/LW |
| 1. Prebiotics in infant nutrition | Exclusion for not probiotic intervention | YL/LW |
| 1. Effects of Butyrate Against Pediatric Obesity: The BAPO randomized-controlled trial | Exclusion for not probiotic intervention | YL/LW |
| 1. Genetic Polymorphisms, Mediterranean Diet and Microbiota-Associated Urolithin Metabotypes can Predict Obesity in Childhood-Adolescence | Exclusion for not probiotic intervention | YL/LW |
| 1. The Safe and Effective Use of Plant-Based Diets with Guidelines for Health Professionals | Exclusion for review article | YL/LW |
| 1. Probiotics for preventing gestational diabetes | Exclusion for not overweight or obesity | YL/LW |
| 1. Probiotics and Maternal Mental Health: A Randomised Controlled Trial among Pregnant Women with Obesity | Exclusion for not children | YL/LW |
| 1. The Effect of Weaning with Adult Food Typical of the Mediterranean Diet on Taste Development and Eating Habits of Children: A Randomized Trial | Exclusion for not probiotic intervention | YL/LW |
| 1. A new possible therapeutic strategy in obesityrelated liver disease: preliminary data with *lactobacillus rhamnosus* strain GG | Exclusion for obesity with other disease | YL/LW |
| 1. Efficacy of metformin and fermentable fiber combination therapy in adolescents with severe obesity and insulin resistance: study protocol for a double-blind randomized controlled trial | Exclusion for not probiotic intervention | YL/LW |
| 1. Safety Assessment of Bacteroides Uniformis CECT 7771, a Symbiont of the Gut Microbiota in Infants | Exclusion for not children | YL/LW |
| 1. Pediatric non-alcoholic fatty liver disease: Recent advances | Exclusion for review article | YL/LW |
| 1. The Stance4Health Project: Evaluating a Smart Personalised Nutrition Service for Gut Microbiota Modulation in Normal-and Overweight Adults and Children with Obesity, Gluten-Related Disorders or Allergy/Intolerance to Cow's Milk | Exclusion for not probiotic intervention | YL/LW |
| 1. Do athletes' responses to coach autonomy support and control depend on the situation and athletes' personal motivation? | Exclusion for not children | YL/LW |
| 1. Perinatal Polyunsaturated Fatty Acid Status and Obesity Risk | Exclusion for not children | YL/LW |
| 1. Serum metabolite profiling yields insights into health promoting effect of *A. muciniphila* in human volunteers with a metabolic syndrome | Exclusion for not children | YL/LW |
| 1. Wheat bran with reduced particle size increases serum SCFAs in obese subjects without improving health parameters compared with a maltodextrin placebo | Exclusion for not probiotic intervention | YL/LW |
| 1. Insight into the prebiotic concept: lessons from an exploratory, double blind intervention study with inulin-type fructans in obese women | Exclusion for not probiotic intervention | YL/LW |
| 1. A critical review of the role of milk and other dairy products in the development of obesity in children and adolescents | Exclusion for review article | YL/LW |
| 1. Microbiota manipulation for weight change | Exclusion for review article | YL/LW |
| 1. Antibiotic exposure and risk of weight gain and obesity: protocol for a systematic review | Exclusion for review protocol | YL/LW |
| 1. Weight Gain and Obesity in Infants and Young Children Exposed to Prolonged Antibiotic Prophylaxis | Exclusion for not probiotic intervention | YL/LW |
| 1. Regular exercise attenuates inflammatory signaling pathways and modulates metabolomic profile in gut microbiota from obese paediatric patients | Exclusion for not probiotic intervention | YL/LW |
| 1. Beneficial effects of aerobic and resistance combined training on inflammatory signaling pathways and gut microbiota in obese pediatric patients | Exclusion for not probiotic intervention | YL/LW |
| 1. Comparison of Ursodeoxycholic Acid to Metformin to treat women with Gestational Diabetes Mellitus | Exclusion for not children | YL/LW |
| 1. Effects of probiotics on nonalcoholic fatty liver disease in obese children and adolescents | Exclusion for obesity with other disease | YL/LW |
| 1. Impact of wheat aleurone on biomarkers of cardiovascular disease, gut microbiota and metabolites in adults with high body mass index: a double-blind, placebo-controlled, randomized clinical trial | Exclusion for not probiotic intervention | YL/LW |
| 1. PHAGE Study: effects of Supplemental Bacteriophage Intake on Inflammation and Gut Microbiota in Healthy Adults | Exclusion for not probiotic intervention | YL/LW |
| 1. Human Milk Oligosaccharides Modulate Fecal Microbiota and Are Safe for Use in Children With Overweight: A Randomized Controlled Trial | Exclusion for not probiotic intervention | YL/LW |
| 1. Late preterm birth has direct and indirect effects on infant gut microbiota development during the first six months of life | Exclusion for not probiotic intervention | YL/LW |
| 1. Body Mass Index and Gut Microbiome: A Cluster-Randomized, Controlled, Pilot Feasibility Study | Exclusion for not probiotic intervention | YL/LW |
| 1. Predictors of becoming overweight among pediatric patients at risk for urinary tract infections | Exclusion for not probiotic intervention | YL/LW |
| 1. Gut microbiome biomarkers in adolescent obesity: A regional study | Exclusion for not probiotic intervention | YL/LW |
| 1. Efficacy of probiotics in non-alcoholic fatty liver disease in adult and children: A meta-analysis of randomized controlled trials | Exclusion for review article | YL/LW |
| 1. Gut microbiota in adolescent girls with polycystic ovary syndrome: effects of randomized treatments | Exclusion for not overweight or obesity | YL/LW |
| 1. Cognitive Function and the Microbiome | Exclusion for not overweight or obesity | YL/LW |
| 1. Systematic Review: Nutrition and Physical Activity in the Management of Paediatric Nonalcoholic Fatty Liver Disease | Exclusion for review article | YL/LW |
| 1. The Benefit of Probiotics in Pediatric Nonalcoholic Fatty Liver Disease: A Meta-analysis of Randomized Control Trials | Exclusion for review article | YL/LW |
| 1. A randomised controlled trial of a duodenal-jejunal bypass sleeve device (EndoBarrier) compared with standard medical therapy for the management of obese subjects with type 2 diabetes mellitus | Exclusion for not probiotic intervention | YL/LW |
| 1. Probiotics to Adolescents With Obesity: Effects on Inflammation and Metabolic Syndrome | Exclusion for no needed data | YL/LW |
| 1. Nutritional Intervention Preconception and During Pregnancy to Maintain Healthy Glucose Metabolism and Offspring Health ("NiPPeR"): study protocol for a randomised controlled trial | Exclusion for not children | YL/LW |
| 1. Improvement of chronic inflammation in obese children consecutive to probiotic intake | Exclusion for obesity with other disease | YL/LW |
| 1. New treatment modalities for obesity | Exclusion for review article | YL/LW |
| 1. Distinct Gut Microbiota in Southeastern African and Northern European Infants | Exclusion for not children | YL/LW |
| 1. Effect of an infant formula containing sn-2 palmitate on fecal microbiota and metabolome profiles of healthy term infants: a randomized, double-blind, parallel, controlled study | Exclusion for not children | YL/LW |
| 1. Consensus and contentious statements on the use of probiotics in clinical practice: A south east Asian gastro-neuro motility association working team report | Exclusion for review article | YL/LW |
| 1. Multistrain probiotic increases the gut microbiota diversity in obese pregnant women: results from a randomized, double-blind placebo-controlled study | Exclusion for not children | YL/LW |
| 1. Effects of probiotics (Vivomixx (R)) in obese pregnant women and their newborn: study protocol for a randomized controlled trial | Exclusion for study protocol | YL/LW |
| 1. Role of androgens in energy metabolism affecting on body composition, metabolic syndrome, type 2 diabetes, cardiovascular disease, and longevity: lessons from a meta-analysis and rodent studies | Exclusion for not probiotic intervention | YL/LW |
| 1. Effect of probiotics and dietary changes on adiposity in children | Exclusion for not only probiotic intervention | LW/LQ |
| 1. Effect of probiotics and dietary changes on inflammatory markers and adiposity in children | Exclusion for not only probiotic intervention | LW/LQ |
| 1. Effect of case study versus video simulation on nursing students' satisfaction, self-confidence, and knowledge: a quasi-experimental study | Exclusion for not children | LW/LQ |
| 1. Link between gut microbiota and health outcomes in inulin -treated obese patients: lessons from the Food4Gut multicenter randomized placebo-controlled trial | Exclusion for not probiotic intervention | LW/LQ |
| 1. Facilitating a high-quality dietary pattern induces shared microbial responses linking diet quality, blood pressure, and microbial sterol metabolism in caregiver-child dyads | Exclusion for not probiotic intervention | LW/LQ |
| 1. Characterization of the Stool Microbiome in Hispanic Preschool Children by Weight Status and Time | Exclusion for not probiotic intervention | LW/LQ |
| 1. Gut microbiota and BMI throughout childhood: the role of *firmicutes*, *bacteroidetes*, and short-chain fatty acid producers | Exclusion for not probiotic intervention | LW/LQ |
| 1. The Impacts of Fish Oil and/or Probiotic Intervention on Low-Grade Inflammation, IGFBP-1 and MMP-8 in Pregnancy: a Randomized, Placebo-Controlled, Double-Blind Clinical Trial | Exclusion for not children | LW/LQ |
| 1. The efficacy of probiotics and/or n-3 long-chain polyunsaturated fatty acids intervention on maternal prenatal and postnatal depressive and anxiety symptoms among overweight and obese women | Exclusion for not children | LW/LQ |
| 1. Prebiotic supplementation improves appetite control in children with overweight and obesity: a randomized controlled trial | Exclusion for not probiotic intervention | LW/LQ |
| 1. Low-protein formula slows weight gain in infants of overweight mothers | Exclusion for not probiotic intervention | LW/LQ |
| 1. Effects of synbiotic on anthropometry, lipid profile and oxidative stress in obese children | Exclusion for not probiotic intervention | LW/LQ |
| 1. The effect of low carbohydrate diet on food addiction and gut microflora | Exclusion for not probiotic intervention | LW/LQ |
| 1. Benefits of repeated individual dietary counselling in long-term weight control in women after delivery | Exclusion for not probiotic intervention | LW/LQ |
| 1. Effects of a Diet Containing Sources of Prebiotics and Probiotics and Modification of the Gut Microbiota on the Reduction of Body Fat | Exclusion for not probiotic intervention | LW/LQ |
| 1. Probiotic and prebiotic interventions for non-alcoholic fatty liver disease: a systematic review and network meta-analysis | Exclusion for review article | LW/LQ |
| 1. Probiotic supplementation increases obesity with no detectable effects on liver fat or gut microbiota in obese Hispanic adolescents: a 16-week, randomized, placebo-controlled trial | Meeting the inclusion criteria for meta-analysis and systematic review | LW/LQ |
| 1. Impact of probiotics during weaning on the metabolic and inflammatory profile: follow-up at school age | Exclusion for not children | LW/LQ |
| 1. Probiotics during weaning: a follow-up study on effects on body composition and metabolic markers at school age | Exclusion for not overweight or obesity | LW/LQ |
| 1. A randomized triple-masked controlled trial on the effects of synbiotics on inflammation markers in overweight children | Exclusion for not probiotic intervention | LW/LQ |
| 1. Inflammation: Depression Fans the Flames and Feasts on the Heat | Exclusion for not overweight or obesity | LW/LQ |
| 1. Effects of synbiotic supplementation on anthropometric measurements, glucose metabolism, lipid parameters in children with exogenous obesity: A randomized, double blind, placebocontrolled clinical trial (probesity-2 trial) | Exclusion for not probiotic intervention | LW/LQ |
| 1. Arabinoxylan oligosaccharides and polyunsaturated fatty acid effects on gut microbiota and metabolic markers in overweight individuals with signs of metabolic syndrome: a randomized cross-over trial | Exclusion for not probiotic intervention | LW/LQ |
| 1. Protocol for a multicentre, parallel, randomised, controlled trial on the effect of sweeteners and sweetness enhancers on health, obesity and safety in overweight adults and children: the SWEET project | Exclusion for study protocol | LW/LQ |
| 1. Concurrent Prebiotic Intake Reverses Insulin Resistance Induced by Early-Life Pulsed Antibiotic in Rats | Exclusion for animal experiment | LW/LQ |
| 1. Effect of alive probiotic on insulin resistance in type 2 diabetes patients: randomized clinical trial | Exclusion for not overweight or obesity | LW/LQ |
| 1. Childhood Obesity: Current Situation and Future Opportunities | Exclusion for review article | LW/LQ |
| 1. The Effects of *Limosilactobacillus reuteri* LR-99 Supplementation on Body Mass Index, Social Communication, Fine Motor Function, and Gut Microbiome Composition in Individuals with Prader-Willi Syndrome: a Randomized Double-Blinded Placebo-Controlled Trial | Exclusion for not overweight or obesity | LW/LQ |
| 1. Impact of bacterial probiotics on obesity, diabetes and non-alcoholic fatty liver disease related variables: a systematic review and meta-analysis of randomised controlled trials | Exclusion for review article | LW/LQ |
| 1. Yogurt consumption during pregnancy and preterm delivery in Mexican women: A prospective analysis of interaction with maternal overweight status | Exclusion for not children | LW/LQ |
| 1. Probiotic *Bifidobacterium* strains and galactooligosaccharides improve intestinal barrier function in obese adults but show no synergism when used together as synbiotics | Exclusion for not children | LW/LQ |
| 1. Consuming yellow pea fiber reduces voluntary energy intake and body fat in overweight/obese adults in a 12-week randomized controlled trial | Exclusion for not probiotic intervention | LW/LQ |
| 1. Predictors of Session Attendance in Intimate Partner Violence Treatment for Trauma-Exposed Veterans | Exclusion for not children | LW/LQ |
| 1. Effect of *Lactobacillus salivarius* Ls-33 on fecal microbiota in obese adolescents | Meeting the inclusion criteria for systematic review but exclusion from the meta-analysis for no needed data | LW/LQ |
| 1. A Synbiotic Mixture of Scgos/Lcfos and *Bifidobacterium Breve* M-16V Is Able to Restore the Delayed Colonization of *Bifidobacterium* Observed in C-Section Delivered Infants | Exclusion for not children | LW/LQ |
| 1. Effects of education and support groups organized by IBCLCs in early postpartum on breastfeeding | Exclusion for not children | LW/LQ |
| 1. Effects of Fecal Microbiome Transfer in Adolescents With Obesity: the Gut Bugs Randomized Controlled Trial | Exclusion for not probiotic intervention | LW/LQ |
| 1. Effects of Inulin-Type Fructans on Appetite, Energy Intake, and Body Weight in Children and Adults: Systematic Review of Randomized Controlled Trials | Exclusion for review article | LW/LQ |
| 1. Effect of oligofructose supplementation on body weight in overweight and obese children: a randomised, double-blind, placebo-controlled trial | Exclusion for not probiotic intervention | LW/LQ |
| 1. A systematic review of microbiome changes and impact of probiotic supplementation in children and adolescents with neuropsychiatric disorders | Exclusion for review article | LW/LQ |
| 1. Study protocol: optimized complementary feeding study (OTIS): a randomized controlled trial of the impact of a protein-reduced complementary diet based on Nordic foods | Exclusion for study protocol | LW/LQ |
| 1. Probiotics in pregnancy and maternal outcomes: a systematic review | Exclusion for review article | LW/LQ |
| 1. Effects of vaginal seeding on gut microbiota, body mass index, and allergy risks in infants born through cesarean delivery: a randomized clinical trial | Exclusion for not probiotic intervention | LW/LQ |
| 1. Long-term safety and efficacy of perinatal probiotic intervention: evidence from a follow-up study of four randomized, double-blind, placebo-controlled trials | Exclusion for not children | LW/LQ |
| 1. The impact of perinatal probiotic intervention on the development of overweight and obesity: follow-up study from birth to 10 years | Exclusion for not randomized controlled trial | LW/LQ |
| 1. Impact of maternal probiotic-supplemented dietary counselling on pregnancy outcome and prenatal and postnatal growth: a double-blind, placebo-controlled study | Exclusion for not children | LW/LQ |
| 1. A pilot study of synbiotic supplementation on breast milk mineral concentrations and growth of exclusively breast fed infants | Exclusion for not children | LW/LQ |
| 1. Evaluations of Lifestyle, Dietary, and Pharmacologic Treatments for Pediatric Nonalcoholic Fatty Liver Disease: A Systematic Review | Exclusion for review article | LW/LQ |
| 1. Effect of maternal supplement beverage with and without probiotics during pregnancy and lactation on maternal and infant health: a randomized controlled trial in the Philippines | Exclusion for not children | LW/LQ |
| 1. Fermented soy product supplemented with isoflavones affected fat depots in juvenile rats | Exclusion for animal experiment | LW/LQ |
| 1. Probiotic therapy outcomes in body composition of children and adolescent with obesity, a nonrandomized controlled trial | Exclusion for not randomized controlled trial | LW/LQ |
| 1. Six weeks of oral Echinacea purpurea supplementation does not enhance the production of serum erythropoietin or erythropoietic status in recreationally active males with above-average aerobic fitness | Exclusion for not children | LW/LQ |
| 1. An alcohol-free beer enriched with isomaltulose and a resistant dextrin modulates gut microbiome in subjects with type 2 diabetes mellitus and overweight or obesity: a pilot study | Exclusion for not probiotic intervention | LW/LQ |
| 1. Impact of dietary fiber supplementation on modulating microbiota-host-metabolic axes in obesity | Exclusion for not probiotic intervention | LW/LQ |
| 1. The Macronutrient Composition of Infant Formula Produces Differences in Gut Microbiota Maturation That Associate with Weight Gain Velocity and Weight Status | Exclusion for not probiotic intervention | LW/LQ |
| 1. Primed to perform: comparing different pre-performance routine interventions to improve accuracy in closed, self-paced motor tasks | Exclusion for not probiotic intervention | LW/LQ |
| 1. Urinary (1)H-NMR-based metabolic profiling of children with NAFLD undergoing VSL#3 treatment | Exclusion for not overweight or obesity | LW/LQ |
| 1. Review article: the management of paediatric nonalcoholic fatty liver disease | Exclusion for review article | LW/LQ |
| 1. Effects of pro-/synbiotic supplementation on anthropometric and metabolic indices in overweight or obese children and adolescents: A systematic review and meta-analysis | Exclusion for review article | LW/LQ |
| 1. The Effects of Probiotic Supplements on Blood Markers of Endotoxin and Lipid Peroxidation in Patients Undergoing Gastric Bypass Surgery; a Randomized, Double-Blind, Placebo-Controlled, Clinical Trial with 13 Months Follow-Up | Exclusion for not overweight or obesity | LW/LQ |
| 1. Metagenomics analysis of gut microbiota in response to diet intervention and gestational diabetes in overweight and obese women: a randomised, double-blind, placebo-controlled clinical trial | Exclusion for not children | LW/LQ |
| 1. Impact of combined consumption of fish oil and probiotics on the serum metabolome in pregnant women with overweight or obesity | Exclusion for not children | LW/LQ |
| 1. A time-restricted feeding intervention in children and adolescents with obesity: The TRansForm study protocol | Exclusion for study protocol | LW/LQ |
| 1. Consumption of stilbenes and flavonoids is linked to reduced risk of obesity independently of fiber intake | Exclusion for not probiotic intervention | LW/LQ |
| 1. Optimise the microbial flora with milk and yoghurt to prevent disease | Exclusion for review article | LW/LQ |
| 1. Probiotics reduce self-reported symptoms of upper respiratory tract infection in overweight and obese adults: should we be considering probiotics during viral pandemics? | Exclusion for not children | LW/LQ |
| 1. Daily probiotic use is associated with a reduced rate of upper respiratory tract symptoms in overweight and obese people | Exclusion for not children | LW/LQ |
| 1. Delivery mode-associated gut microbiota in the first 3 months of life in a country with high obesity rates: A descriptive study | Exclusion for not randomized controlled trial | LW/LQ |
| 1. Obesity, Diabetes, and Gut Microbiota The hygiene hypothesis expanded? | Exclusion for review article | LW/LQ |
| 1. The Effects of Synbiotic Supplementation on Antioxidant Capacity and Arm Volumes in Survivors of Breast Cancer-Related Lymphedema | Exclusion for not probiotic intervention | LW/LQ |
| 1. The Effect of Probiotics on Low-grade Inflammation, Microbiota and Risk Factors for Metabolic Syndrome in Obese Children | Exclusion for no full text | LW/LQ |
| 1. Effects of Probiotics in Obese Children | Exclusion for no full text | LW/LQ |
| 1. Effect of Prebiotic Intake on Adiposity, Satiety and Gut Microbiota in Overweight and Obese Children | Exclusion for not probiotic intervention | LW/LQ |
| 1. Potential Role of n-3 Fatty Acids in the Treatment of NAFLD in Pediatric Patients | Exclusion for not probiotic intervention | LW/LQ |
| 1. Growth and Metabolism in Infants Fed Protein-reduced, Alpha-lactalbumin Enriched Formula | Exclusion for not children | LW/LQ |
| 1. Effect of Probiotics (Vivomixx®) on Weight, Microbiota and Glucose Tolerance in Obese Pregnant Women and Their Newborn | Exclusion for not children | LW/LQ |
| 1. Transplantation of Microbes for Treatment of Metabolic Syndrome & NAFLD | Exclusion for not probiotic intervention | LW/LQ |
| 1. Preventing Atopic Dermatitis and ALLergies in Children | Exclusion for not overweight or obesity | LW/LQ |
| 1. The Effect of *Lactobacillus Reuteri* ATCC PTA 6475 on Volumetric Bone Mineral Density in Patients With Osteopenia | Exclusion for not overweight or obesity | LW/LQ |
| 1. Healthy Eating Through Reduction Of Excess Sugar | Exclusion for not probiotic intervention | LW/LQ |
| 1. Effects of HMOs on the Faecal Microbiota and on Host Metabolism in Obese Children | Exclusion for not probiotic intervention | LW/LQ |
| 1. The Effects of Butyrate on Children With Obesity | Exclusion for not probiotic intervention | LW/LQ |
| 1. Vaginal Microbiome Seeding and Health Outcomes in Cesarean-delivered Neonates | Exclusion for not children | LW/LQ |
| 1. BIFI-OBESE: clinical Trial in Paediatric Obesity | Exclusion for no full text | LW/LQ |
| 1. Effects of Almond Consumption on Cardiovascular, Metabolomic, and Microbiome Profiles in Millennials | Exclusion for not probiotic intervention | LW/LQ |
| 1. Composition and Collection Feasibility of Gut Microbiota in Children With and Without Obesity | Exclusion for not probiotic intervention | LW/LQ |
| 1. Fit Child - Clinical Study on Weight Control | Exclusion for no full text | LW/LQ |
| 1. Gut Microbiome, Adiposity, and Probiotics (GMAP) | Exclusion for no full text | LW/LQ |
| 1. Dietary Management of Gestational Diabetes in Obese Pregnant Women | Exclusion for not children | LW/LQ |
| 1. Response of the Gut Microbiome and Circulating Metabolome to Diet in Children: ancillary Study to KIDFIT (NCT03405246) | Exclusion for no full text | LW/LQ |
| 1. Targeting the Gut Microbiome for Prader-Willi Syndrome Treatment | Exclusion for not overweight or obesity | LW/LQ |
| 1. Motivational Intervention on the Gut Microbiota of Obese Children | Exclusion for not probiotic intervention | LW/LQ |
| 1. Strategies To OPpose Sugars With Non-nutritive Sweeteners Or Water (STOP Sugars NOW) Trial | Exclusion for not probiotic intervention | LW/LQ |
| 1. Impact of Dietary Fiber as Prebiotics on Intestinal Microbiota in Obese Thai Children | Exclusion for not probiotic intervention | LW/LQ |
| 1. Evaluating the Metabolic Effects of Polylactose: a Novel Prebiotic | Exclusion for not probiotic intervention | LW/LQ |
| 1. Impact of *B. Bifidum* 900791 Intake on Breast Milk Characteristics of Obese Mothers | Exclusion for not children | LW/LQ |
| 1. Effect of a Three Combined Probiotics Supplementation on Weight Loss in Obese/Overweight Children | Exclusion for no full text | LW/LQ |
| 1. Effect of Liraglutide on Microbiome in Obesity | Exclusion for not probiotic intervention | LW/LQ |
| 1. Fiber Intervention on Gut Microbiota in Children With Prader-Willi Syndrome | Exclusion for not probiotic intervention | LW/LQ |
| 1. China Heart Diet for People With High Cardiovascular Risk in China (DECIDE-Diet) | Exclusion for not overweight or obesity | LW/LQ |
| 1. *L. Reuteri* DSM 17938 and *L. Reuteri* ATCC PTA 6475 in Moderate to Severe Irritable Bowel in Adults | Exclusion for not children | LQ/YL |
| 1. Assessment of the Impact of Type of Probiotic, Delivery Type and Feeding Type on Baby's Microbiota After Dysbiotic Delivery | Exclusion for not children | LQ/YL |
| 1. Fiber and Metformin Combination Therapy in Adolescents With Severe Obesity and Insulin Resistance | Exclusion for not only probiotic intervention | LQ/YL |
| 1. Maternal Betaine Supplementation During Breastfeeding | Exclusion for not children | LQ/YL |
| 1. Effects of Butyrate Against Pediatric Obesity | Exclusion for not probiotic intervention | LQ/YL |
| 1. Probiotic Treatment for Prader-Willi Syndrome | Exclusion for not overweight or obesity | LQ/YL |
| 1. PROBESITY-2: synbiotics in Pediatric Obesity | Exclusion for not probiotic intervention | LQ/YL |
| 1. Childhood Obesity Microbiome Study | Exclusion for no full text | LQ/YL |
| 1. The Influence of GNiib® in Obesity Management in Obese Young Individuals in Hong Kong | Exclusion for no full text | LQ/YL |
| 1. Dietary Intervention With Probiotic Pasta and Evaluation of the Effects on Metabolic and Inflammatory Status | Exclusion for no full text | LQ/YL |
| 1. Dietary Modulation of Gut Microbiota in Overweight/Obese Adolescents and COVID-19 Infection | Exclusion for obesity with other disease | LQ/YL |
| 1. Time-Restricted Feeding in Children and Adolescents With Obesity | Exclusion for not probiotic intervention | LQ/YL |
| 1. Growth, Allergy and Neurodevelopment in Infants on Hydrolysed Formula | Exclusion for not children | LQ/YL |
| 1. Immune-supportive Diet and Gut Permeability in Allergic Children | Exclusion for not overweight or obesity | LQ/YL |
| 1. Comparison of Plant-based or Animal-based Protein on Anthropocentric and Metabolic Parameters in Obese Subjects | Exclusion for not probiotic intervention | LQ/YL |
| 1. Assessment of the Effects of Synbiotic on Gut Microbiota Composition in Scholars With Overweight | Exclusion for not probiotic intervention | LQ/YL |
| 1. Keiki (Pediatric) Produce Prescription (KPRx) Program Hawaii | Exclusion for no full text | LQ/YL |
| 1. Full-fat Dairy Products, Body Weight Control and Metabolic Health | Exclusion for no full text | LQ/YL |
| 1. Comparative Clinical Study Between Empagliflozin Versus Pioglitazone in Non-diabetic Patients With Non-alcoholic Steatohepatitis | Exclusion for not probiotic intervention | LQ/YL |
| 1. Prebiotics Reduce Body Fat and Alter Intestinal Microbiota in Children Who Are Overweight or With Obesity | Exclusion for not probiotic intervention | LQ/YL |
| 1. Effect of prebiotic fiber-induced changes in gut microbiota on adiposity in obese and overweight children | Exclusion for not probiotic intervention | LQ/YL |
| 1. Effect of prebiotic fiber intake on adiposity and inflammation in overweight and obese children: assessing the role of the gut microbiota | Exclusion for not probiotic intervention | LQ/YL |
| 1. SPRING: an RCT study of probiotics in the prevention of gestational diabetes mellitus in overweight and obese women | Exclusion for not children | LQ/YL |
| 1. Nonalcoholic Fatty Liver Disease A Challenge for Pediatricians | Exclusion for not overweight or obesity | LQ/YL |
| 1. Fighting Fatty Liver Diseases with Nutritional Interventions, Probiotics, Symbiotics, and Fecal Microbiota Transplantation (FMT) | Exclusion for not overweight or obesity | LQ/YL |
| 1. Effect of antenatal dietary interventions in maternal obesity on pregnancy weight-gain and birthweight: healthy Mums and Babies (HUMBA) randomized trial | Exclusion for not children | LQ/YL |
| 1. Correction: a randomised controlled demonstration trial of multifaceted nutritional intervention and or probiotics: the healthy mums and babies (HUMBA) trial [BMC Pregnancy Childbirth., 16, (2016) (373)] DOI: 10.1186/s12884-016-1149-8 | Exclusion for not children | LQ/YL |
| 1. Effect of Commonly Used Pediatric Antibiotics on Gut Microbial Diversity in Preschool Children in Burkina Faso: A Randomized Clinical Trial | Exclusion for not probiotic intervention | LQ/YL |
| 1. Probiotic supplementation attenuates increases in body mass and fat mass during high-fat diet in healthy young adults | Exclusion for not children | LQ/YL |
| 1. A review of the pathogenic and therapeutic role of nutrition in pediatric nonalcoholic fatty liver disease | Exclusion for review article | LQ/YL |
| 1. Gut-liver axis and probiotics: Their role in non-alcoholic fatty liver disease | Exclusion for not overweight or obesity | LQ/YL |
| 1. Evaluation of differential effects of metformin treatment in obese children according to pubertal stage and genetic variations: study protocol for a randomized controlled trial | Exclusion for study protocol | LQ/YL |
| 1. Evaluation of the gut microbiota after metformin intervention in children with obesity: a metagenomic study of a randomized controlled trial | Exclusion for review article | LQ/YL |
| 1. Gut Microbiota and Obesity in Adults and Children: The State of the Art | Exclusion for review article | LQ/YL |
| 1. Safety and tolerability of experimental hookworm infection in humans with metabolic disease: study protocol for a phase 1b randomised controlled clinical trial | Exclusion for study protocol | LQ/YL |
| 1. Gut microbiota (GM) and gut-liver axis (GLA) components in obesity related NAFLD: a pediatric pilot study | Exclusion for obesity with other disease | LQ/YL |
| 1. Effects of School-Based Participation Program to Prevent Multiple Risk Behaviors in Thai Male Adolescents | Exclusion for not overweight or obesity | LQ/YL |
| 1. Mechanisms of Action of Probiotics | Exclusion for review article | LQ/YL |
| 1. The effects of myo-inositol and probiotic supplementation in a high-fat-fed preclinical model of glucose intolerance in pregnancy | Exclusion for not children | LQ/YL |
| 1. Antibiotic exposure in the first three years of life and weight gain during childhood | Exclusion for not probiotic intervention | LQ/YL |
| 1. Effect of Synbiotic Supplementation in Children with Nonalcoholic Fatty Liver Disease: a Randomized Controlled Trial | Exclusion for not probiotic intervention | LQ/YL |
| 1. Environmental Determinants of Type 1 Diabetes: From Association to Proving Causality | Exclusion for not overweight or obesity | LQ/YL |
| 1. Exercise training modulates the gut microbiota profile and impairs inflammatory signaling pathways in obese children | Exclusion for not probiotic intervention | LQ/YL |
| 1. Effect of probiotic *Lactobacillus plantarum* Dad-13 powder consumption on the gut microbiota and intestinal health of overweight adults | Exclusion for not children | LQ/YL |
| 1. Effects of *bifidobacterium animalis subsp. lactis* on children with Prader-Willi syndrome: a randomized, double-blind, placebo-controlled, crossover trial | Exclusion for not overweight or obesity | LQ/YL |
| 1. The study protocol for a pseudo-randomised pre-post designed controlled intervention trial to study the effects of a 7-week cooking program on self-efficacy and biomarkers of health: the ECU lifestyle and biomarkers get connected study (ECULABJMOF) including the Jamie's Ministry of Food WA participant experience | Exclusion for study protocol | LQ/YL |
| 1. Use of hydrolysates and amino acid formulas in infancy - dietary management under modified conditions | Exclusion for not children | LQ/YL |
| 1. Clinical application of a cocoa and unripe banana flour beverage for overweight women with abdominal obesity: Prospective, double-blinded and randomized clinical trial | Exclusion for not children | LQ/YL |
| 1. Improved Plasma Lipids, Anti-Inflammatory Activity, and Microbiome Shifts in Overweight Participants: two Clinical Studies on Oral Supplementation with Algal Sulfated Polysaccharide | Exclusion for not probiotic intervention | LQ/YL |
| 1. Effects of probiotics combined with dietary and lifestyle modification on clinical, biochemical, and radiological parameters in obese children with nonalcoholic fatty liver disease/nonalcoholic steatohepatitis: a randomized clinical trial | Exclusion for obesity with other disease | LQ/YL |
| 1. IS THERE A RELATIONSHIP BETWEEN GUT MICROBIOTA, PROBIOTICS AND BODY WEIGHT MODULATION? | Exclusion for review article | LQ/YL |
| 1. The effects of synbiotic supplementation on some cardio-metabolic risk factors in overweight and obese children: a randomized triple-masked controlled trial | Exclusion for not only probiotic intervention | LQ/YL |
| 1. Inulin-type fructans modulate intestinal Bifidobacterium species populations and decrease fecal short-chain fatty acids in obese women | Exclusion for not probiotic intervention | LQ/YL |
| 1. Reinforcement of intestinal epithelial barrier by arabinoxylans in overweight and obese subjects: a randomized controlled trial: arabinoxylans in gut barrier | Exclusion for not probiotic intervention | LQ/YL |
| 1. Effects of a Diet-Based Weight-Reducing Program with Probiotic Supplementation on Satiety Efficiency, Eating Behaviour Traits, and Psychosocial Behaviours in Obese Individuals | Exclusion for not children | LQ/YL |
| 1. *Bifidobacterium pseudocatenulatum* CECT 7765 supplementation improves inflammatory status in insulin-resistant obese children | Exclusion for obesity with other disease | LQ/YL |
| 1. Understanding the role of gut microbes and probiotics in obesity: How far are we? | Exclusion for review article | LQ/YL |
| 1. Fish Oil And/Or Probiotics Intervention in Overweight/Obese Pregnant Women and Overweight Risk in 24-Month-Old Children | Exclusion for not children | LQ/YL |
| 1. Early Gut Fungal and Bacterial Microbiota and Childhood Growth | Exclusion for not overweight or obesity | LQ/YL |
| 1. Tenth year reenrollment randomized trial investigating the effects of childhood probiotics and calcium supplementation on height and weight at adolescence | Exclusion for not only probiotic intervention | LQ/YL |
| 1. Soy food intake associates with changes in the metabolome and reduced blood pressure in a gut microbiota dependent manner | Exclusion for not probiotic intervention | LQ/YL |
| 1. Prospective Longitudinal Trends in Body Composition and Clinical Outcomes 3 Years Following Sleeve Gastrectomy | Exclusion for not probiotic intervention | LQ/YL |
| 1. Gut microbiota and obesity: an overview of microbiota to microbial-based therapies | Exclusion for review article | LQ/YL |
| 1. Multiomic Predictors of Short-Term Weight Loss and Clinical Outcomes During a Behavioral-Based Weight Loss Intervention | Exclusion for not children | LQ/YL |
| 1. Biotechnological Applications of Probiotics: A Multifarious Weapon to Disease and Metabolic Abnormality | Exclusion for review article | LQ/YL |
| 1. Microbial transmission from mothers with obesity or diabetes to infants: an innovative opportunity to interrupt a vicious cycle | Exclusion for review article | LQ/YL |
| 1. Supplementation with *Bifidobacterium breve* BR03 and B632 strains improved insulin sensitivity in children and adolescents with obesity in a cross-over, randomized double-blind placebo-controlled trial | Exclusion for obesity with other disease | LQ/YL |
| 1. Efficacy of the treatment with *bifidobacterium breve* b632 and *bifidobacterium breve* br03 on endocrine response to the oral glucose tolerance test in pediatric obesity: a cross-over double blind randomized controlled trial | Exclusion for obesity with other disease | LQ/YL |
| 1. Efficacy of diode and CO2 lasers along with calcium and fluoride-containing compounds for the remineralization of primary teeth | Exclusion for not overweight or obesity | LQ/YL |
| 1. Probiotics in Adolescent Prediabetes: A Pilot RCT on Glycemic Control and Intestinal Bacteriome | Exclusion for no needed data | LQ/YL |
| 1. A maternal higher-complex carbohydrate diet increases *bifidobacteria* and alters early life acquisition of the infant microbiome in women with gestational diabetes mellitus | Exclusion for not probiotic intervention | LQ/YL |
| 1. The Effectiveness of the Young–Old Link and Growth Intergenerational Program in Reducing Age Stereotypes | Exclusion for not overweight or obesity | LQ/YL |
| 1. Effects of Low-Carbohydrate Diet and Exercise Training on Gut Microbiota | Exclusion for not probiotic intervention | LQ/YL |
| 1. The effect of synbiotic supplementation on anthropometric indices, appetite, and constipation in people with hypothyroidism: a randomized, double-blind, placebo-controlled trial | Exclusion for not probiotic intervention | LQ/YL |
| 1. Impact of diet on the human intestinal microbiota | Exclusion for not probiotic intervention | LQ/YL |
| 1. Effects of Complementary Feeding With Different Protein-Rich Foods on Infant Growth and Gut Health: study Protocol | Exclusion for study protocol | LQ/YL |
| 1. Obesity and severe asthma | Exclusion for not overweight or obesity | LQ/YL |
| 1. Three-year follow-up of a randomised controlled trial to reduce excessive weight gain in the first two years of life: protocol for the POI follow-up study | Exclusion for study protocol | LQ/YL |
| 1. Evaluation of the effect of *Lactobacillus reuteri* V3401 on biomarkers of inflammation, cardiovascular risk and liver steatosis in obese adults with metabolic syndrome: a randomized clinical trial (PROSIR) | Exclusion for not children | LQ/YL |
| 1. A concise review of non-alcoholic fatty liver disease | Exclusion for review article | LQ/YL |
| 1. Functional response to a microbial synbiotic in the gastrointestinal system of children: a randomized clinical trial | Exclusion for not probiotic intervention | LQ/YL |
| 1. Structural Alteration of Gut Microbiota during the Amelioration of Human Type 2 Diabetes with Hyperlipidemia by Metformin and a Traditional Chinese Herbal Formula: a Multicenter, Randomized, Open Label Clinical Trial | Exclusion for not probiotic intervention | LQ/YL |
| 1. The Effects of Human Milk Oligosaccharide Supplementation During Critical Periods of Development on the Mesolimbic Dopamine System | Exclusion for not probiotic intervention | LQ/YL |
| 1. The effects of inulin supplementation on insulin resistance and branched-chain amino acids in paediatric obesity: a randomised, double-blinded, placebo-controlled trial | Exclusion for not probiotic intervention | LQ/YL |
| 1. Calorie restriction and synbiotics effect on quality of life and edema reduction in breast cancer-related lymphedema, a clinical trial | Exclusion for not overweight or obesity | LQ/YL |
| 1. Weight status and dietary intake determine serum leptin concentrations in pregnant and lactating women and their infants | Exclusion for not probiotic intervention | LQ/YL |
| 1. The impact of probiotic supplementation during pregnancy on DNA methylation of obesity-related genes in mothers and their children | Exclusion for not children | LQ/YL |
| 1. Therapeutic options in pediatric non alcoholic fatty liver disease: current status and future directions | Exclusion for review article | LQ/YL |
| 1. Effects of *Lactobacillus rhamnosus* strain GG in pediatric obesity-related liver disease | Exclusion for obesity with other disease | LQ/YL |
| 1. Probiotics: an update | Exclusion for review article | LQ/YL |
| 1. Role of Dietary Factors, Food Habits, and Lifestyle in Childhood Obesity Development: A Position Paper From the European Society for Paediatric Gastroenterology, Hepatology and Nutrition Committee on Nutrition | Exclusion for review article | LQ/YL |
| 1. Docosahexaenoic acid and non-alcoholic fatty liver disease in obese children: a novel approach? | Exclusion for not overweight or obesity | LQ/YL |
| 1. A randomized double-blind placebo controlled pilot study of probiotics in adolescents with severe obesity | Exclusion for obesity with other disease | LQ/YL |
| 1. Impact of probiotics during weaning on the metabolic and inflammatory profile: follow-up at school age | Exclusion for not a probiotic supplement for overweight or obese children | LQ/YL |
| 1. Probiotics during weaning: a follow-up study on effects on body composition and metabolic markers at school age | Exclusion for not a probiotic supplement for overweight or obese children | LQ/YL |
| 1. Effects of inulin supplementation on body composition and metabolic outcomes in children with obesity | Exclusion for not probiotic intervention | LQ/YL |
| 1. The Impact of Dietary Fiber as a Prebiotic on Inflammation in Children with Obesity | Exclusion for not probiotic intervention | LQ/YL |
| 1. Effects of inulin supplementation on gut microbiota in obese children: a randomised, doubleblinded placebo-controlled study | Exclusion for not probiotic intervention | LQ/YL |
| 1. Metagenomic Insights into the Degradation of Resistant Starch by Human Gut Microbiota | Exclusion for not overweight or obesity | LQ/YL |
| 1. Role-play versus lecture methods in community health volunteers | Exclusion for not overweight or obesity | LQ/YL |
| 1. Effects of fungal beta-glucans on health - a systematic review of randomized controlled trials | Exclusion for review article | YL/LW |
| 1. Effect of Probiotic Supplementation on Newborn Birth Weight for Mother with Gestational Diabetes Mellitus or Overweight/Obesity: A Systematic Review and Meta-Analysis | Exclusion for review article | YL/LW |
| 1. Effects of oligosaccharide-sialic acid (OS) compound on maternal-newborn gut microbiome, glucose metabolism and systematic immunity in pregnancy: protocol for a randomised controlled study | Exclusion for study protocol | YL/LW |
| 1. Strain engraftment competition and functional augmentation in a multi-donor fecal microbiota transplantation trial for obesity | Exclusion for not probiotic intervention | YL/LW |
| 1. Oral administration of maternal vaginal microbes at birth to restore gut microbiome development in infants born by caesarean section: A pilot randomised placebo-controlled trial | Exclusion for not children | YL/LW |
| 1. Genomic microdiversity of Bifidobacterium pseudocatenulatum underlying differential strain-level responses to dietary carbohydrate intervention | Exclusion for not overweight or obesity | YL/LW |
| 1. Role of Probiotics in Non-alcoholic Fatty Liver Disease: Does Gut Microbiota Matter? | Exclusion for not overweight or obesity | YL/LW |
| 1. Mendelian Randomization Analysis Reveals Causal Effects of the Human Gut Microbiota on Abdominal Obesity | Exclusion for not probiotic intervention | YL/LW |
| 1. The mediating effect of coping styles and self‐efficacy between perceived stress and satisfaction with QOL in Chinese adolescents with type 1 diabetes | Exclusion for not overweight or obesity | YL/LW |
| 1. Non-Alcoholic Fatty Liver Disease in Children: Focus on Nutritional Interventions | Exclusion for not overweight or obesity | YL/LW |
| 1. Gut microbiota and pediatric obesity/non-alcoholic fatty liver disease | Exclusion for review article | YL/LW |
| 1. Greater alteration of gut microbiota occurs in childhood obesity than adulthood obesity | Exclusion for not probiotic intervention | YL/LW |
| 1. A Quasi-Trial Investigation of an In-Service Training to Improve Social Workers' Professional Competence in China | Exclusion for not overweight or obesity | YL/LW |
| 1. Metabolic phenotypes and the gut microbiota in response to dietary resistant starch type 2 in normal-weight subjects: a randomized crossover trial | Exclusion for not overweight or obesity | YL/LW |
| 1. Evaluation of a WeChat‐based life review programme for cancer patients: a quasi‐experimental study | Exclusion for not overweight or obesity | YL/LW |
| 1. Probiotics Can Further Reduce Waist Circumference in Adults with Morbid Obesity after Bariatric Surgery: A Systematic Review and Meta-Analysis of Randomized Controlled Trials | Exclusion for review article | YL/LW |
| 317. Impact of probiotics supplement on the gut microbiota in neonates with antibiotic exposure: an open-label single-center randomized parallel controlled study | Exclusion for not children | YL/LW |
